# Cooperative Assembly of 2D‐MOF Nanoplatelets into Hierarchical Carpets and Tubular Superstructures for Advanced Air Filtration

**DOI:** 10.1002/anie.202117730

**Published:** 2022-03-29

**Authors:** Friedrich Schwotzer, Jacob Horak, Irena Senkovska, Elke Schade, Tatiana E. Gorelik, Philipp Wollmann, Mai Lê Anh, Michael Ruck, Ute Kaiser, Inez M. Weidinger, Stefan Kaskel

**Affiliations:** ^1^ Inorganic Chemistry Center I Technische Universität Dresden Bergstr. 66 01069 Dresden Germany; ^2^ IWS Dresden Winterbergstr. 28 01277 Dresden Germany; ^3^ Electron Microscopy Group of Materials Science (EMMS) Central Facility for Electron Microscopy Ulm University Albert-Einstein-Allee 11 89081 Ulm Germany; ^4^ Electrochemistry Technische Universität Dresden Zellescher Weg 19 01069 Dresden Germany; ^5^ Inorganic Chemistry II Technische Universität Dresden Bergstr. 66 01069 Dresden Germany; ^6^ Max Planck Institute for Chemical Physics of Solids Nöthnitzer Str. 40 01187 Dresden Germany

**Keywords:** 2D Material, Air Filtration, Chemisorption, Mesocrystal, Metal–Organic Frameworks, Self-Assembly

## Abstract

Clean air is an indispensable prerequisite for human health. The capture of small toxic molecules requires the development of advanced materials for air filtration. Two‐dimensional nanomaterials offer highly accessible surface areas but for real‐world applications their assembly into well‐defined hierarchical mesostructures is essential. DUT‐134(Cu) ([Cu_2_(dttc)_2_]_*n*_, dttc=dithieno[3,2‐*b* : 2′,3′‐*d*]thiophene‐2,6‐dicarboxylate]) is a metal–organic framework forming platelet‐shaped particles, that can be organized into complex structures, such as millimeter large free‐standing layers (carpets) and tubes. The structured material demonstrates enhanced accessibility of open metal sites and significantly enhanced H_2_S adsorption capacity in gas filtering tests compared with traditional bulk analogues.

## Graphical abstract

A 2D copper‐based metal–organic framework, DUT‐134(Cu), self‐assembles into carpet‐like mesocrystals offering enhanced accessibility of open metal sites for air‐filtration applications.

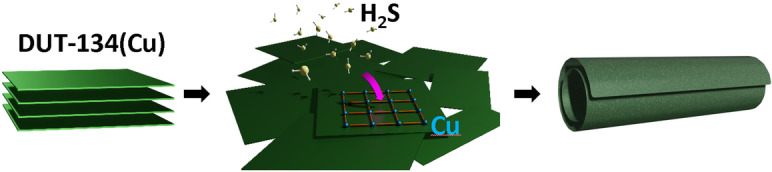

## Introduction

Metal–organic frameworks are porous coordination networks constructed from metal clusters and bridging organic ligands.^[1]^ The majority of these materials form three‐dimensional structures, but a variety of layered and pillared layer MOFs is also known.^[2]^ Due to their modular structure, these networks can be deliberately designed for specific applications and therefore provide an enormous potential for many technological areas.^[3]^ MOFs are considered as versatile materials benefitting from their regularly ordered metal sites and organic moieties (crystallinity) and could therefore achieve breakthroughs especially in areas such as energy storage, catalysis, separation or sensing.[^4^, ^5^, ^6^]

The morphology of a MOF crystal is influenced by the intrinsic nature of the solid and relates to anisotropy of chemical bonding and connectivity. However, the control of crystallites shape, including synthesis of anisotropic 2D particles, is essential for process integration of MOFs. Variations in material dimensionality, on his part, are expected to influence the surface interaction, chemical reactivity, catalytic activity, electronic band structure etc.^[7]^ The deliberate synthesis of anisotropically figured MOF crystals is not trivial. Nevertheless, many great advancements have been made in this field^[8]^ during recent years mainly due to the increasing scientific interest in advanced synthetic 2D materials beyond graphene.[^9^, ^10^] One subclass of MOFs, ideally suited for synthesis of materials anisotropic in shape, are intrinsic 2D MOFs, consisting of layers formed by coordination bonds and held together by weak intermolecular interactions. The prime examples of layered MOF structures are MOFs based on paddle wheel clusters (such as [M_2_(bdc)_2_]_*n*_,[^11^, ^12^] bdc=1,4‐benzene dicarboxylate or [M_4_(TCPP)_2_]_*n*_,^[13]^ TCPP=tetrakis(4‐carboxyphenyl)porphyrin, M=Zn, Cu, Pd) or MOFs based on hexasubstituted triphenylene ligands (for example [Ni_3_(HITP)_2_]_*n*_,^[14]^ (HITP=2,3,6,7,10,11‐hexaiminotriphenylene or [Cu_3_(HHTP)_2_],^[15]^ HHTP=2,3,6,7,10,11‐hexahydroxytriphenylene). Existing synthesis strategies to achieve 2D materials can be classified as top‐down or bottom‐up approaches. The former involves the delamination of bulk MOF crystals; while the latter method is based on the direct synthesis of high‐aspect‐ratio crystals from metal node precursors and organic ligands.[^4^, ^6^]

Although, the synthesis of ultra‐thin 2D layers via the top‐down method is often easier to implement, control of plate thickness and hierarchical assembly is challenging.^[16]^ Here, we focus on the bottom‐up strategy to obtain layers with spatially extended lateral dimensions.

Inspired by bio‐mineralization, enabling the construction of hierarchical architectures with elaborate properties,^[17]^ we focus on self‐organization of MOF platelets into complex superstructures (mesocrystals). The deliberate assembly of MOF‐particles into larger architectures leads to the next generation of functional MOF‐based materials exhibiting sophisticated optical, chemical and mechanical properties.^[18]^ Such an approach may stimulate the implementation of MOFs in catalysis, separation, as photonic crystals, or as metamaterials.[^9^, ^19^] It can also lead towards advanced synthesis of MOF‐based membranes and coatings as a subsequent evolutionary step enhancing the pore accessibility and guest transport kinetics^[20]^ as well as provide fundamental insights useful for (nano)science in general.^[21]^

**Figure 1:**
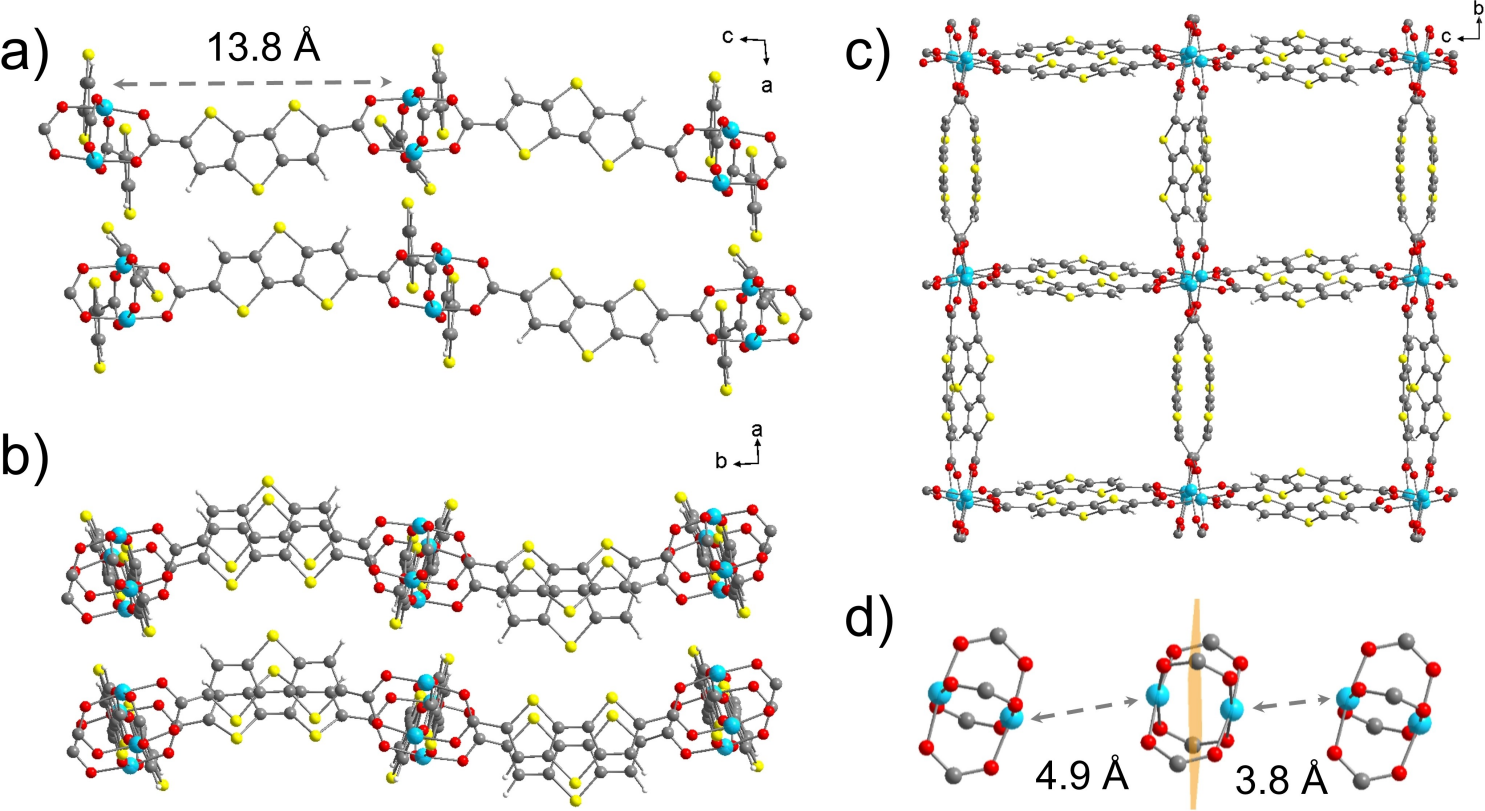
View of the crystal structure of desolvated DUT‐134(Cu) along three crystallographic directions (a–c). Distance between the Cu atoms of the adjoined layers (d). The distances are given between the atom centres. The orange plane represents the plane of the layer. Colour code: C (grey), O (red), S (yellow), Cu (cyan), H (white).

DUT‐134(Cu) is an ideal 2D model system, a typical representative of layered MOFs based on paddle wheel as secondary building unit (Figure 1).^[22]^ Copper paddle wheels interlinked by the ditopic dithieno[3,2‐*b* : 2′,3′‐*d*]thiophene‐2,6‐dicarboxylate (dttc^2−^) linker form undulated layers with the (4,4)‐net structure. The axial positions of the paddle wheels in DUT‐134(Cu)⋅DMF are coordinated by *N,N*‐dimethylformamide (DMF) molecules, resulting in a framework with overall composition [Cu_2_(dttc)_2_(DMF)_2_(L)_*x*_]_*n*_, (L represents the solvent in the pores in addition to the solvent coordinated to the paddle wheels) (Figure S3, Supporting Information). The layers are stacked in AB sequence along the [100] crystallographic direction.The distance between the neighboring layers is 8.8 Å. Upon desolvation a structural transformation reduces the interlayer distance to 6.1 Å resulting in an AA stacking (Figure 1).^[22]^ This structural transformation leads to effective shielding of the axial positions of the paddle wheels (open metal sites), making them hardly accessible for catalytic applications or chemisorptive air filtration. The inaccessibility of planar surfaces in 2D materials due to packing is a key challenge considering 2D materials for real world applications. Despite such layers can be delaminated in suspensions with thickness down to 4–12 nm, for air filtration applications stable hierarchical assemblies are required.

In the following, we describe the synthesis of 2D mesocrystal‐like superstructures obtained by self‐organization of the DUT‐134⋅DMF platelets into large, free‐standing membrane‐like assemblies, in the following termed “carpets”. The resulting semi‐transparent carpets show improved accessibility of the open Cu^2+^‐sites after solvent removal, boosting H_2_S sorption capacity. The direct comparison between the powdered DUT‐134 bulk material and the DUT‐134 carpets shows clear advantages of anisotropically formed assemblies versus the bulk counterpart.

## Results and Discussion

### Synthesis and Carpet Formation

A quasi‐interfacial synthetic approach was applied to synthesize platelets of two‐dimensional DUT‐134⋅DMF for subsequent hierarchical assembly. For the bottom‐up approach, H_2_dttc (as linker) and Cu(NO_3_)_2_⋅3 H_2_O (as a metal precursor) were dissolved in a mixture of DMSO and DMF. A buffer solution of the pure solvent mixture was superimposed on the linker‐containing solution, before adding the fluid containing metal ions. Nucleation and initial growth of crystallites are initiated by the slow diffusion of precursors into the buffer layer.

**Figure 2:**
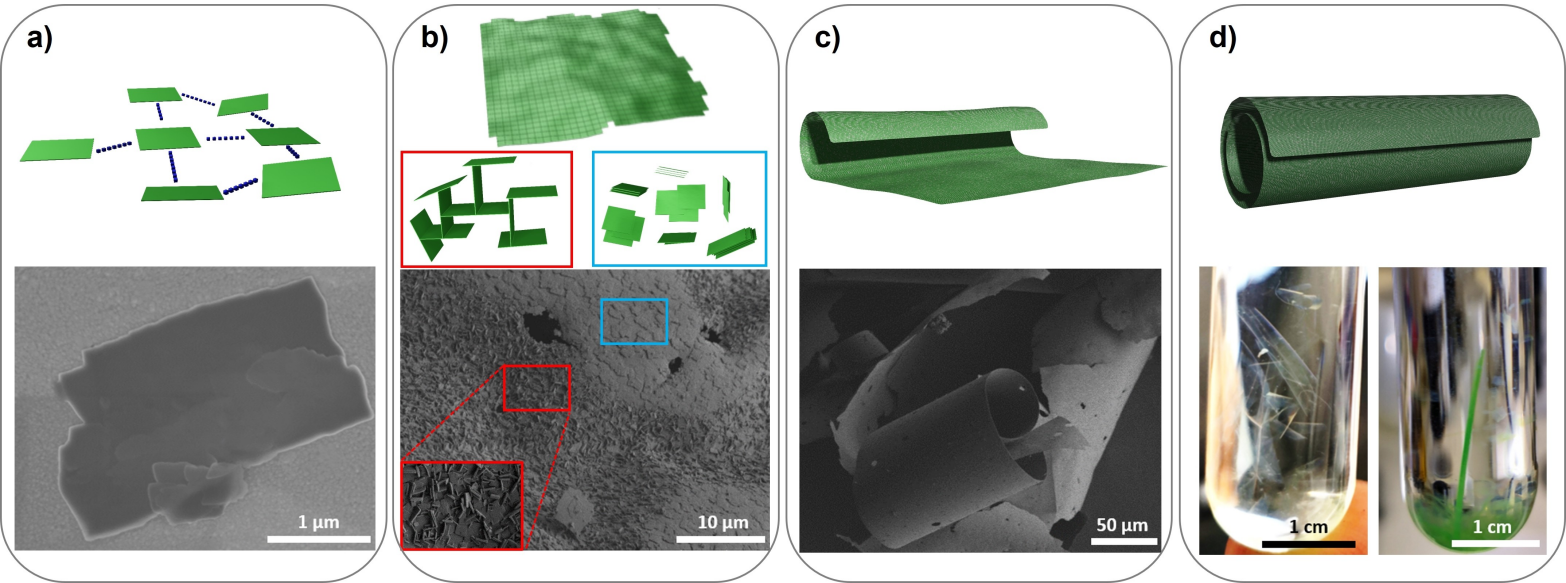
Schematic representation (top) and images (bottom) supporting the carpet formation mechanism: a) formation of separated, anisotropically shaped DUT‐134(Cu)⋅DMF crystallites; b) spontaneous self‐assembly of the crystallites in fresh DMF solution as face to face or edge to face alignment; c, d) free‐standing carpets as well as spontaneous roll up, leading to the formation of macro‐tubes with a diameter of up to ca. 100 μm and a length of up to 20 mm or longer. Bottom: SEM image of a) DUT‐134(Cu) platelets; b) the carpet surface, c) macroscopic structure of the dried carpets. Photographs of: d) semi‐transparent DUT‐134⋅DMF carpet in DMF (left) and large “multi‐walled” macro‐tubes in DMF (right).

The approach resulted in the formation of separated, platelet‐like small crystallites with lateral dimensions of 1–2 μm (Figure 2a). The as‐synthesized platelets were separated from the mother liquor and washed to obtain the starting material for self‐assembly experiments. Subsequently, the platelets were placed into the tube, containing DMF, and incubated at room temperature. After several days, up to 20 mm long, semi‐transparent free‐standing layers (carpets) are formed by spontaneous, cooperative self‐assembly of platelets (Figure 2b–d; Figure S4, Supporting Information). The bulk DUT‐134(Cu)⋅DMF material as well as carpets demonstrate remarkable stability against water (Figure S9b, Supporting Information).

**Figure 3:**
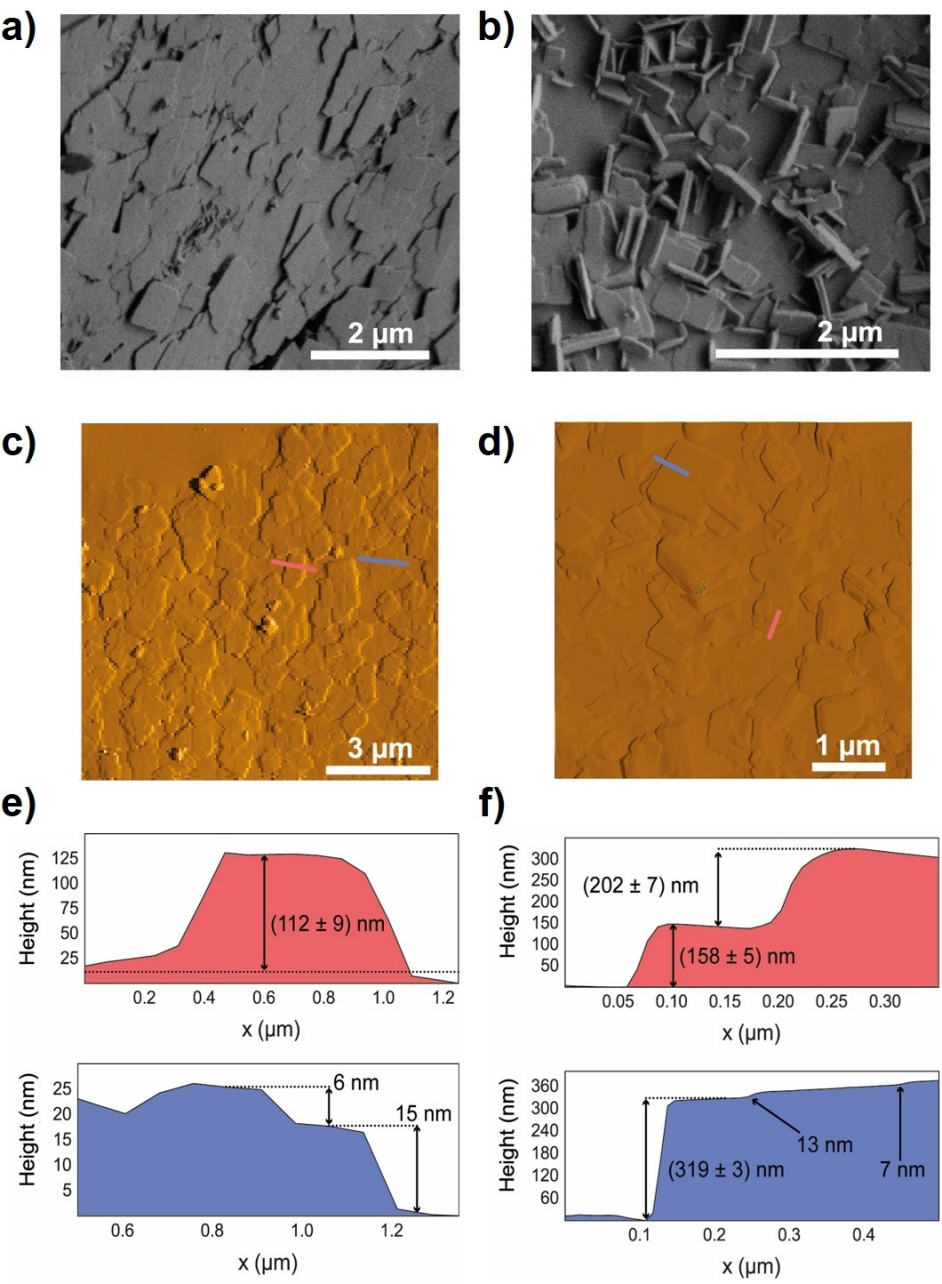
SEM (a, b) and AFM (c, d) images of two different regions of the carpets. The corresponding AFM height profiles are shown in (e) and (f).

SEM images (Figure 3a, b) and AFM analysis (Figure S20, Supporting Information) reveal that small platelet‐like crystallites form extended carpet superstructures with lateral dimensions of several hundred microns and thickness about 0.2–10 μm. The particles in the carpet are aligned in two different manners: edge‐to‐face or face‐to‐face (as depicted in Figure 2b and Figure 3).^[23]^

Atomic force microscopy (AFM) analysis of two different surface domains gives the lateral dimensions for most carpet building particles between 0.5–2.0 μm with a thickness of 6–150 nm (Figure 3c, d).

**Figure 4:**
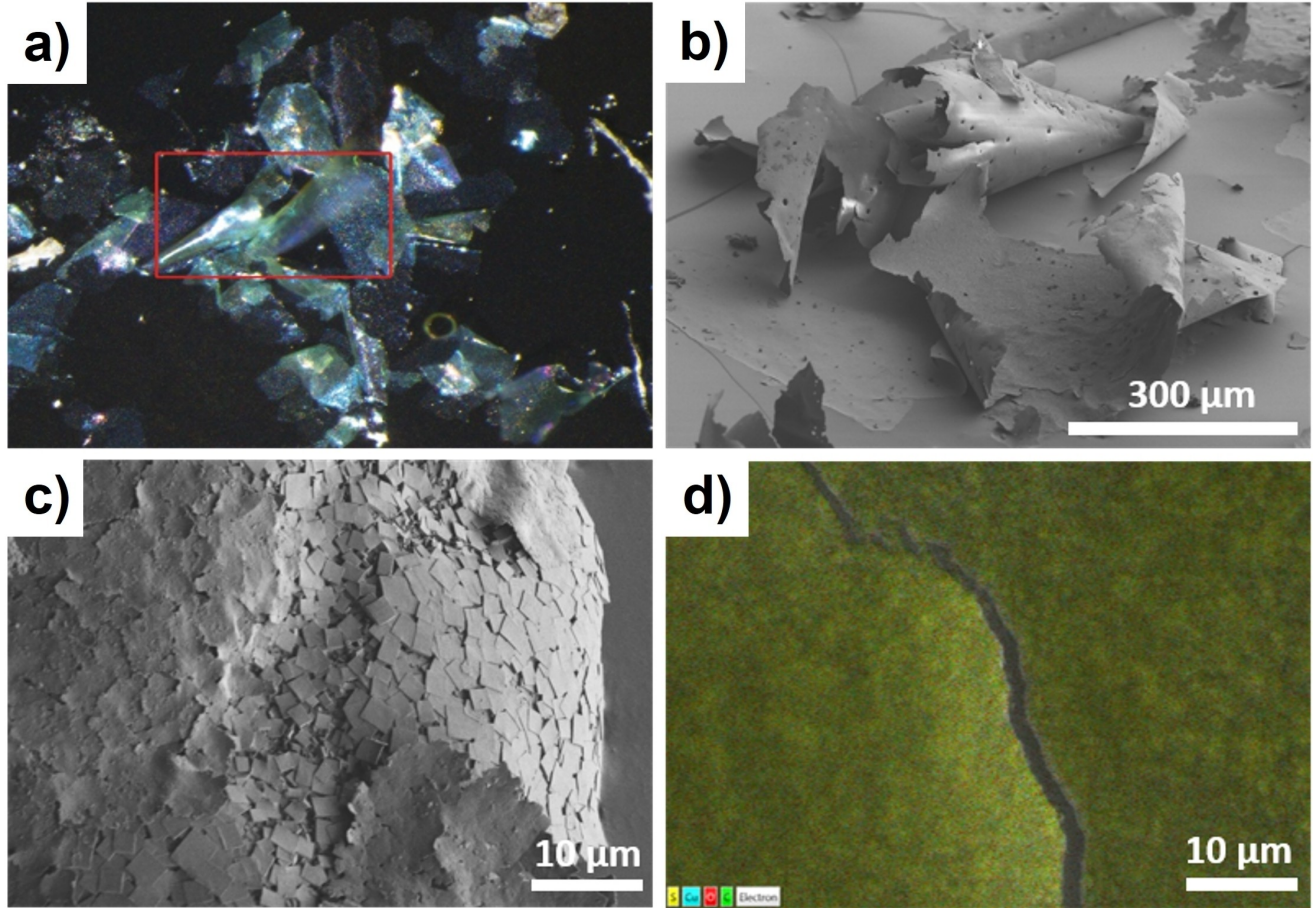
Microscopic images of DUT‐134(Cu) carpets superstructure: a) optical microscope image of supercritical dried sample; b, c) SEM images; and d) SEM with energy dispersive X‐ray spectroscopy (EDS) map. Cu is shown in blue, S in yellow, O in red and C in green. The experimentally determined S : Cu element ratio of 75 : 25 is in perfect agreement with the theoretical ratio.

The Cu : S ratio in the carpets (1 : 3) is in good agreement with the expected composition of DUT‐134, as shown by energy‐dispersive X‐ray spectroscopy (EDS) (Figure 4d; Figure S5, Supporting Information). The Raman spectra of the carpets (Figure S8, S9, Supporting Information) are in good agreement with the spectra of bulk DUT‐134(Cu), confirming the preservation of their chemical nature.

Since the validation of the carpet's crystallographic structure by powder X‐ray diffraction was difficult mainly due to the tiny dimensions of crystallites and the associated peak broadening, as well as the preferred orientation of the platelets, transmission electron microscopy (TEM) and electron diffraction (ED) were used to investigate the supercritically dried samples.

**Figure 5:**
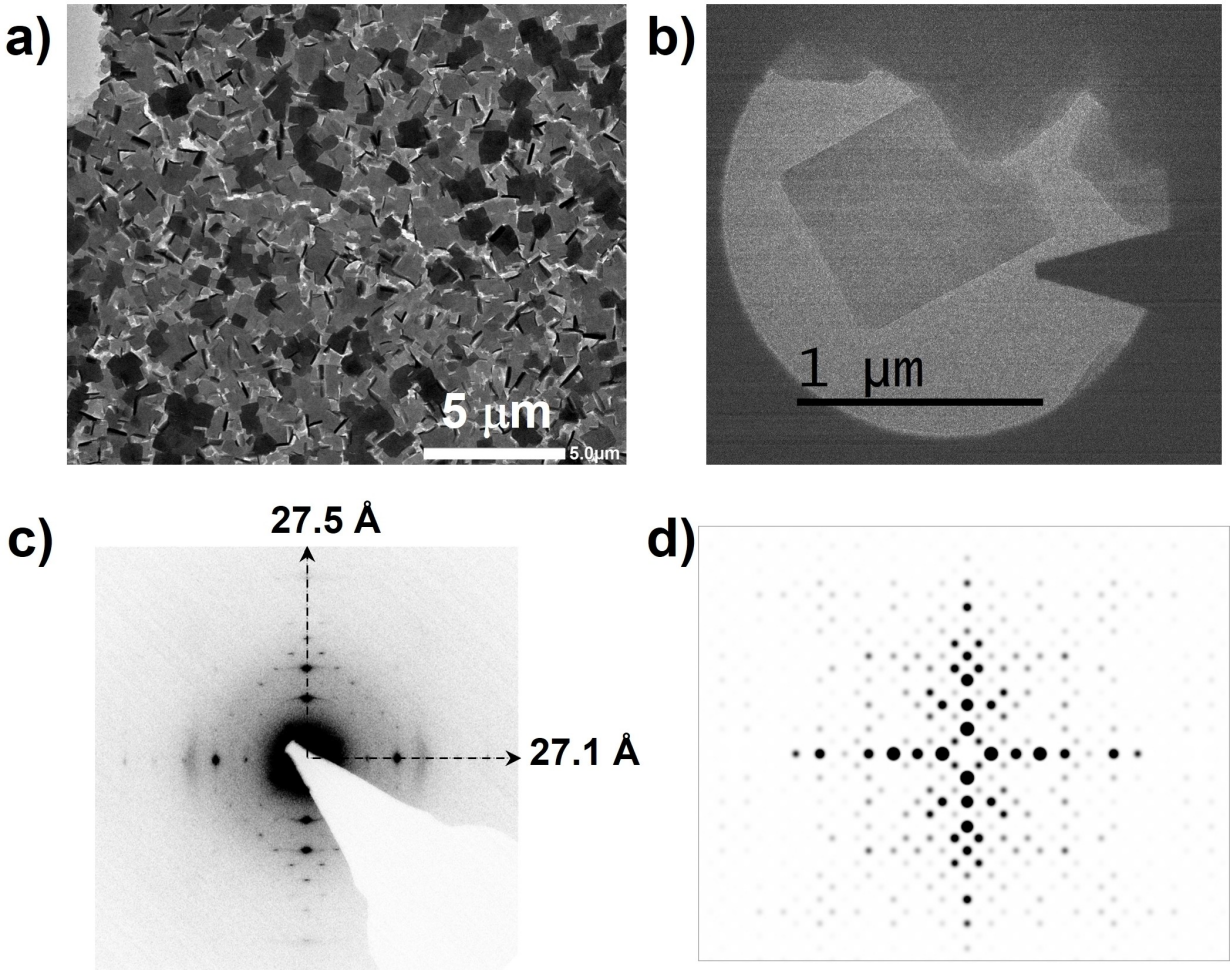
a) TEM image of mesostructure of DUT‐134(Cu) carpet; b) TEM image of the single crystallite as a carpet's building block; c) ED pattern of one [100] zone from one crystal from the carpet‐like assembly (shown in b); d) simulated [100] zone for DUT‐134.

The particle assembly in the carpet is clearly visible in the TEM image (Figure 5a). The ED pattern of [100] zone, obtained from a single crystallite of the carpet (Figure 5c), confirms the structural integrity of the material. The solvent‐free DUT‐134 crystallizes in a monoclinic space group *P*2_1_ */m* with unit cell parameters *a*=13.309 Å, *b*=26.831 Å, *c*=28.169 Å, and *β*=96.59°.^[22]^ The values obtained from the experimental electron diffraction pattern of the [100] zone are 27.5 and 27.1 Å and are thus in reasonable agreement. The intensities of reflections differ from the simulated (Figure 5d), mainly due to the electron beam induced structural damage.

Despite the exact formation mechanism of the carpets from the suspension of platelets may require further investigations, we propose the following assembly mechanism: Crystallites obtained in quasi‐interfacial synthesis attach to the glass walls of the reaction tube, acting as substrate. The DMF supported assembly leads to the formation of the films, which detach from the glass surface easily to form freestanding carpets. DMF may play a special role in the formation of the carpet‐like structure and guides the assembly process, since other solvents, such as EtOH, acetone, 2‐propanol, or MeCN were not suited to support the formation of similar 2D architectures.

In general, the strategies to achieve 2D (nano)crystal self‐assembly usually rely on tuning the repulsive and attractive interactions of the terminal ligands attached to the particle surface.^[24]^ In our case, the van der Waals interaction between DMF molecules (also playing important role in the stacking on the layers into a 3D crystal structure ( Figure S3, Supporting Information)^[22]^ are unique and necessary for carpet formation, acting as an “adhesive” for the particle‐particle association into the carpet‐like structure.

Although van der Waals interactions are in general non‐directional, in the case of 2D MOFs, the anisotropy of the crystal structure introduces regions with different physicochemical properties and intrinsic particle site‐specific interactions, allowing direction‐dependent attraction or repulsion over the surface. The easy exchange of the surface ligands in the MOF chemistry opens a potential way to a “lock‐unlock” mechanism for particle assembly‐disassembly, controlled by the surface ligand chemistry.

**Figure 6:**
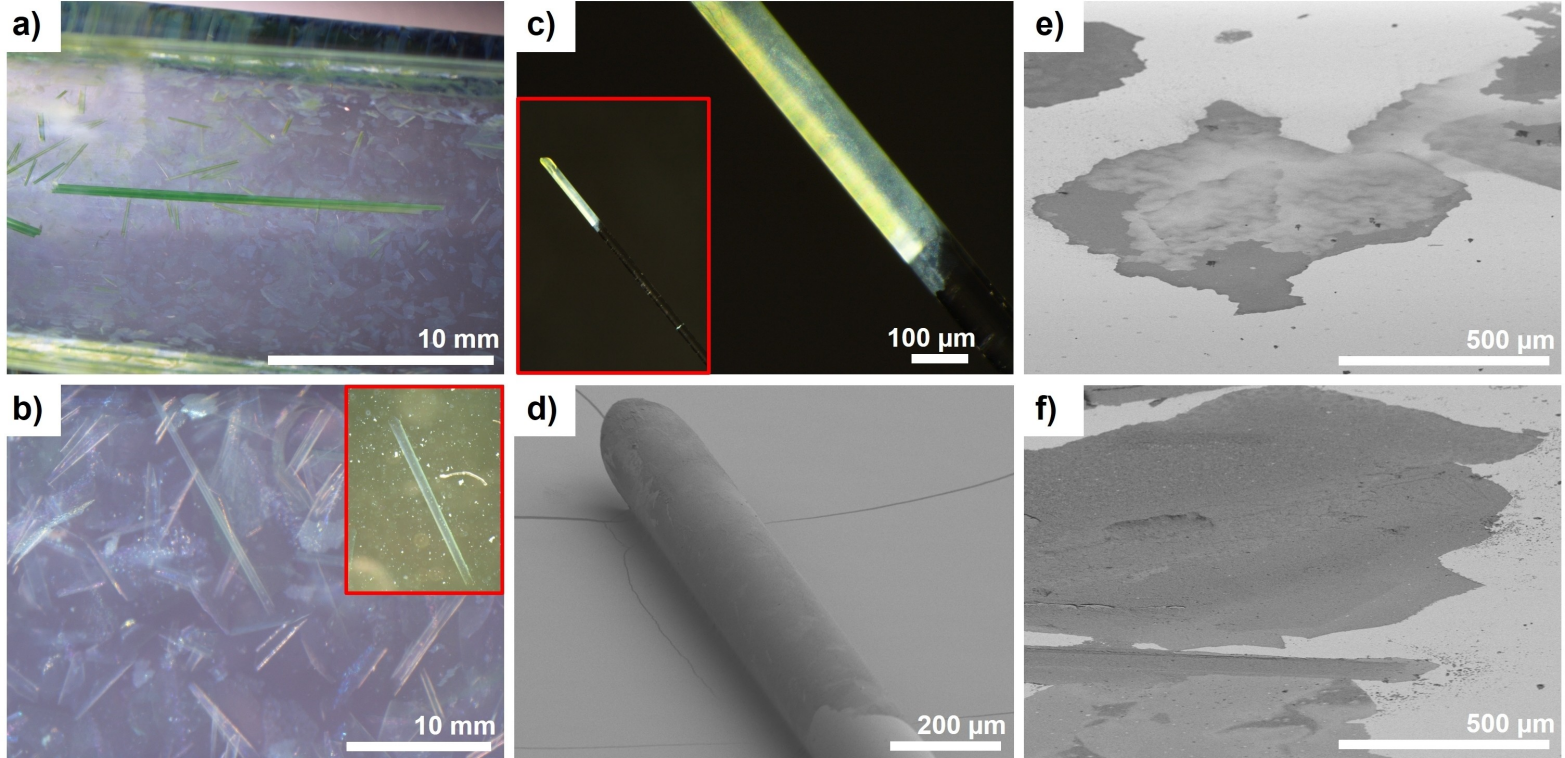
a–c) Light microscopic images of DUT‐134(Cu) carpets. a, b) Carpets in DMF; c) glass capillary with a diameter of 0.1 mm coated with the DUT‐134 layer; d–f) SEM images: d) glass capillary (shown in c) coated with a MOF layer. Carpets deposited on Au‐substrate (e) and Si‐wafer (f) by spin coating.

The synthesized carpets are prone to spontaneous curling with time (within several days), forming macro‐scrolls up to ca. 100 μm in diameter and up to 20 mm in length (Figure 2d, Figure 6b), rendering it, to the best of our knowledge, the first example of bottom‐up macro‐tubular self‐assembly based on MOF materials. The tubules are shape‐persistent and mechanically robust in solution. The aging of the layers in DMF for weeks leads to the formation of “multi‐walled” tubules up to 20 mm in length or even longer (Figure 2d, Figure 6a). Through further growth and roll‐up of the carpets, the once semi‐transparent sheets become gradually darker green in color approaching the color of the stacked 3D bulk material (Figure S10, Supporting Information).

### Carpet Assembly on Planar Surfaces

The synthesis of microscale structures with well‐defined size and shape is an important aspect in material science. However, fabrication of higher‐order structures remains a challenge even for (amorphous) compounds formed by covalent bonds, such as classical polymers.^[25]^ The fabrication of nanostructure assemblies formed from crystalline coordination polymers is even more complex, but has attracted increasing attention in recent years. In addition, the fabrication of coatings and membranes is an important aspect in porous materials based separation technology, making the processing of the crystalline materials into supported membrane architectures very attractive.

The DUT‐134⋅DMF carpets can be easily transferred from the solvent to the planar support (for example on glass, silica, gold, or polymer surfaces) by simple spin coating of the carpet dispersion (Figure 6e, f and Figure S6, S7, Supporting Information). Moreover, the carpets are mechanically stable enough, as well as flexible enough to be folded into 3D macroscopic objects with more complex shapes.

In the first attempts, the surface of the glass capillary, with a diameter of 0.1 mm could be successfully coated by DUT‐134(Cu)⋅DMF carpets via direct transfer of the free‐standing macro‐tube from DMF. The layer perfectly covers the carrier without forming any wrinkles or cracks, leading to a homogeneous coating obtained by wet processing (Figure 6c, d and Figure S6, Supporting Information).

### Air Filtration

**Figure 7:**
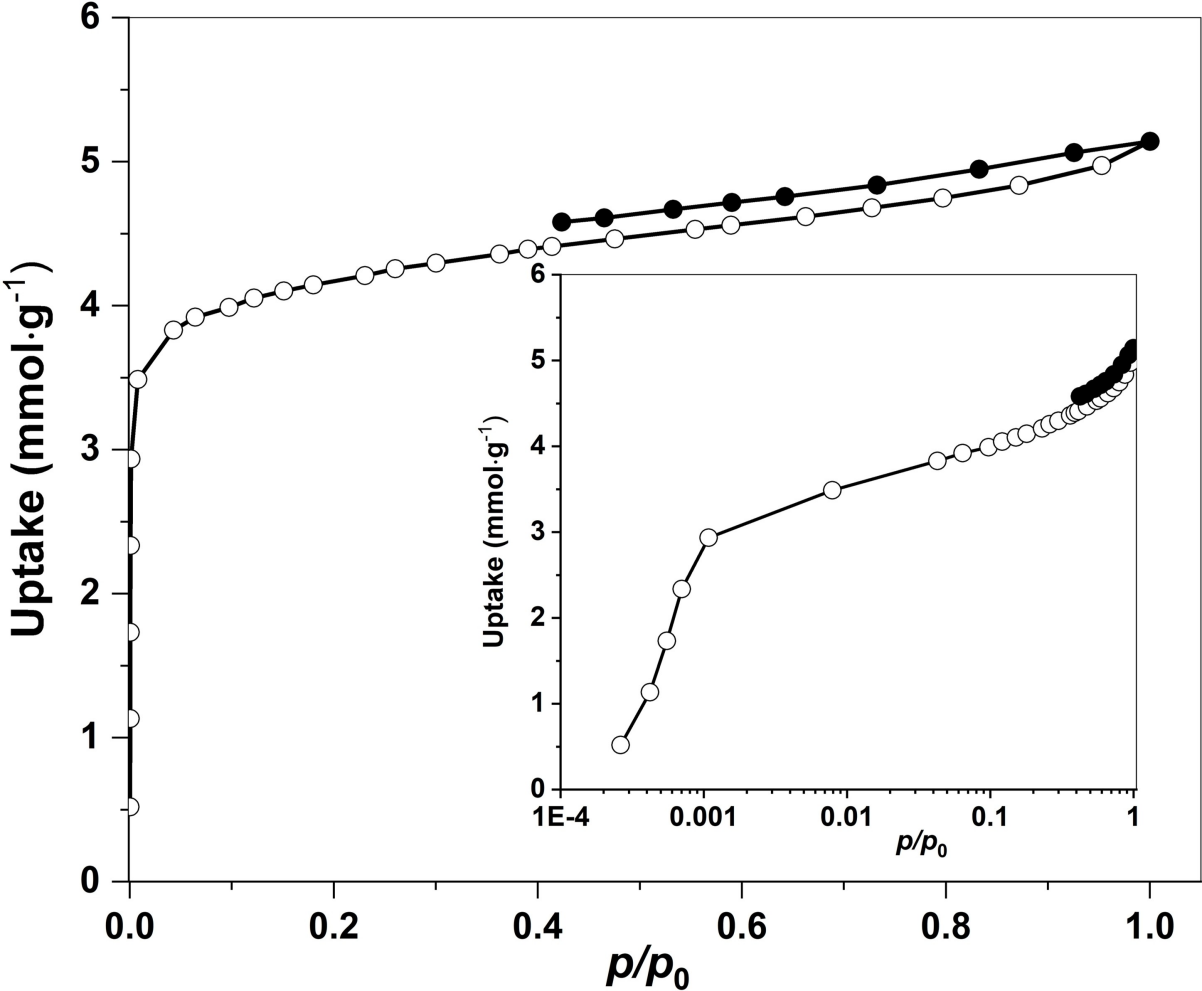
Krypton physisorption isotherm measured at 77 K, *p*_0_=0.224 kPa. Inlet shows the semi‐logarithmic plot. Empty symbols=adsorption, solid symbols=desorption.

Supercritically dried DUT‐134(Cu) bulk crystals offer a specific BET area of 1200 m^2^ g^−1^ and a pore volume of 0.54 cm^3^ g^−1^ (as derived from nitrogen adsorption isotherms measured at 77 K).^[22]^ Adsorption characteristics of the dried carpets are accessible via krypton adsorption, a probe suitable for the investigation of small amounts of material or materials with an extremely small surface area. For krypton physisorption at 77 K, due to the extremely low saturation pressure of 0.35 kPa, the number of molecules in the free space of the sample cell can be significantly reduced, which enables the high sensitivity of the krypton measurement.^[26]^ Due to the small thickness of the carpets and the low packing density, the experiments could be performed using 1 mg of the sample only, with still tolerable error margin. The measured adsorption isotherm confirms the integrity of the MOF resembling a type I isotherm, typical for microporous materials (Figure 7).

In order to demonstrate the advantages of the hierarchical superstructure in comparison to the bulk material with regard to the accessibility of open metal sites, both materials were studied in a dynamic filter test for H_2_S capture. As a benchmark for the H_2_S breakthrough studies, HKUST‐1 was used (0.1 vol.% H_2_S in N_2_) showing an uptake of 2.6 mmol H_2_S per gram accompanied by a color change from deep blue to almost black. This value is close to adsorption capacities reported for HKUST‐1 in the literature (2.7 mmol g^−1^, 0.1 vol.% H_2_S in moist air; 250 mL min^−1^ flow).^[27]^ The theoretical maximum uptake for HKUST‐1 was predicted even higher (19.6 mmol g^−1^)^[28]^ but this value is experimentally not achieved due to decomposition and side reactions leading to inaccessible Cu sites.

DUT‐134 bulk crystals measured under comparable conditions (0.1 vol.% H_2_S in N_2_ at room temperature) show an adsorption capacity of 2.53 mmol g^−1^, which is higher than the value of 1.7 mmol g^−1^ reported for pillared layer [Cu_2_(1,4‐bdc)_2_(dabco)]_*n*_ (1,4‐bdc=benzene‐1,4‐dicarboxylate, dabco=1,4‐diazabicyclo[2.2.2]octane) compound (1 vol % H_2_S in CO_2_/He, 30 mL min^−1^, 298 K).^[29]^ The capacity drops in a subsequent cycle to 0.76 mmol g^−1^ and is almost constant in the third cycle. Such characteristic is typical for irreversible chemisorption on Cu centers in the first cycle, and in subsequent cycles physisorption causes the uptake. The chemisorbed amount is 0.6 mol H_2_S per 1 mol Cu.

**Figure 8:**
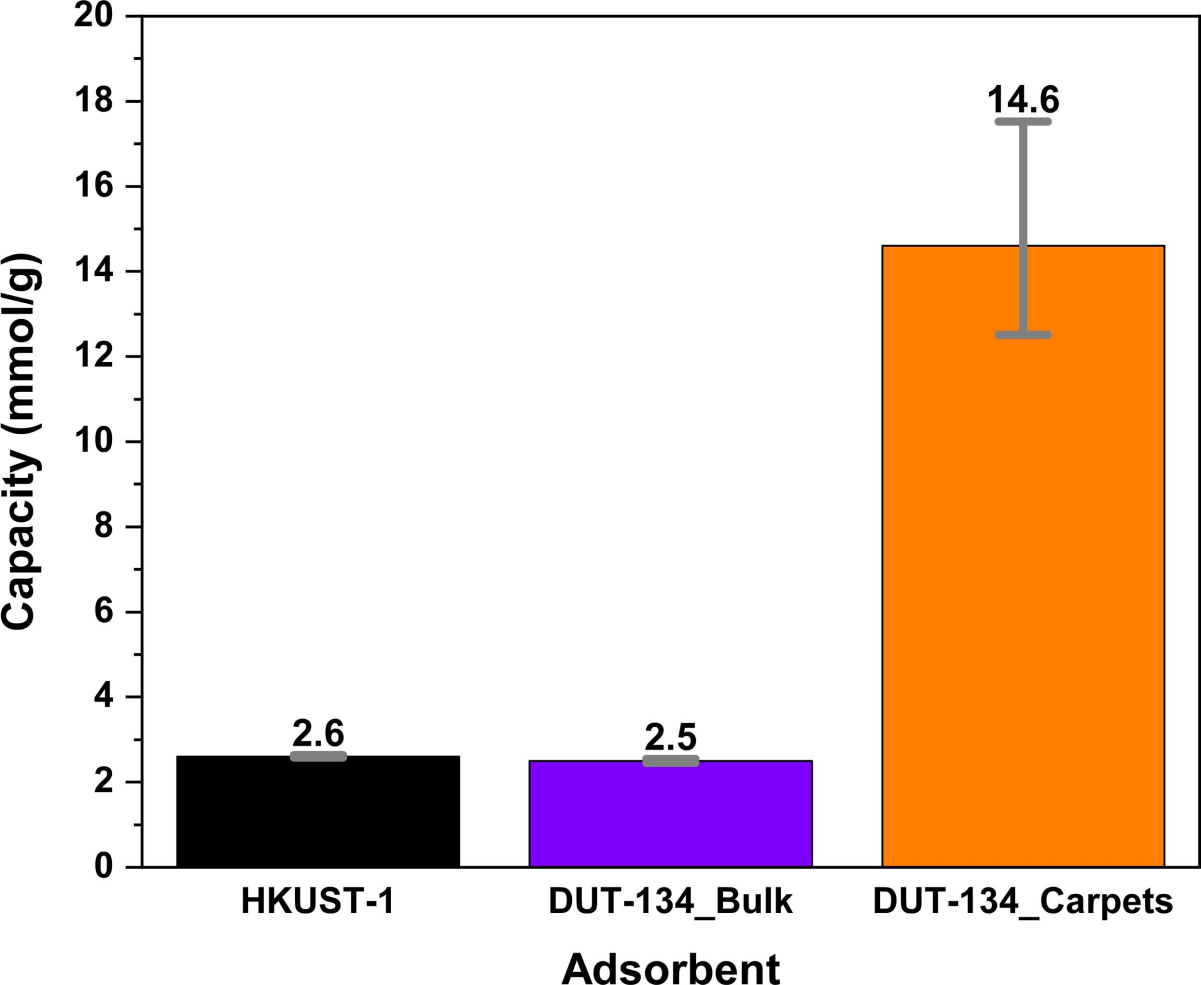
Comparison of H_2_S adsorption capacities at room temperature (Flow rate 380 mL min^−1^, 0.1 vol.% H_2_S in N_2_).

For DUT‐134(Cu) based carpets, however, a remarkably high value of 14.6 mmol g^−1^ could be achieved, almost six times higher than that of the bulk DUT‐134 (Figure 8). The results were also validated by InfraSORP^[30]^ measurements (for more details see Supporting Information). This value is approaching the capacity of MIL‐53(Al)‐TDC, a benchmark with the highest H_2_S adsorption (18.1 mmol g^−1^)^[31]^ ever reported for any adsorbent, but measured at a higher concentration of 5 vol % H_2_S in N_2_ at 303 K. CPO‐27(Ni) (12 mmol g^−1^ at 1 bar H_2_S at 303 K)^[32]^ is clearly outperformed. Hence, our new results demonstrate the significant advantage of the hierarchical carpet architectures against crystalline powders. A potential mechanism behind the chemisorption is polysulfide formation catalysed by the Cu centers of DUT‐134 causing the high uptake of carpets.^[33]^ Cu atoms are not fully accessible in the bulk material due to the structure of the desolvated MOF (Figure 1 and Figure S3, Supporting Information). In contrast, the high external surface area of the carpets and consequently improved accessibility of the Cu sites could be a reasonable explanation for the increased H_2_S uptake.

## Conclusion

The interfacial synthesis approach was successfully used to synthesize platelet‐shaped particles of layered DUT‐134(Cu)⋅DMF ([Cu_2_(dttc)_2_(DMF)_2_(L)_*x*_]_*n*_, dttc: dithieno[3,2‐*b* : 2′,3′‐*d*]thiophene‐2,6‐dicarboxylate, L ‐ solvent) MOF. The solvent‐assisted assembly of the particles facilitates the formation of millimeter‐sized, 2D freestanding mesostructured layers (carpets). The carpets can easily be transferred from the solvent to planar or curved substrates. Incubation of the carpets in the solvent leads to the formation of tubes, composed of rolled MOF sheets. The sheets and tubes could be supercritically desolvated, providing porous materials with a vast number of surface exposed open metal sites accessible for chemisorption. Hence, the H_2_S filtering ability demonstrated in dynamic adsorption experiments, is almost factor 6 higher than that of the corresponding bulk macro crystals. Compared to traditional bulk crystals, the hierarchical assembly of 2D structured MOF materials into carpet‐like architectures offers significant benefits for air‐filtration applications.

## Conflict of interest

The authors declare no conflict of interest.

1

## Supporting information

As a service to our authors and readers, this journal provides supporting information supplied by the authors. Such materials are peer reviewed and may be re‐organized for online delivery, but are not copy‐edited or typeset. Technical support issues arising from supporting information (other than missing files) should be addressed to the authors.

Supporting Information

## Data Availability

All data are shared as electronic Supporting Information upon publication.
