# Supplementary material for: Cooperative Assembly of 2D‐MOF Nanoplatelets into Hierarchical Carpets and Tubular Superstructures for Advanced Air Filtration
Source: Angew Chem Int Ed Engl. 2022 Mar 29;61(22):e202117730. doi: 10.1002/anie.202117730 (PMC9315001; doi:10.1002/anie.202117730)
Supplement: Supplementary file 1 — Supporting Information [file ANIE-61-0-s001.pdf]

Angewandte Chemie
Eine Zeitschrift der Gesellschaft Deutscher Chemiker

Supporting Information

Cooperative Assembly of 2D-MOF Nanoplatelets into Hierarchical Carpets and Tubular Superstructures for Advanced Air Filtration

F. Schwotzer, J. Horak, I. Senkovska, E. Schade, T. E. Gorelik, P. Wollmann, M. L. Anh, M. Ruck, U. Kaiser, I. M. Weidinger, S. Kaskel**

Supporting Information
©Wiley-VCH 2021
69451 Weinheim, Germany

Cooperative Assembly of 2D-MOF Nanoplatelets into Hierarchical Carpets and Tubular Superstructures for Advanced Air Filtration

Friedrich Schwotzer,[a] Jacob Horak,[a] Irena Senkovska,*[a] Elke Schade,[b] Tatiana E. Gorelik,[c] Philipp Wollmann,[d] Mai Lê Anh,[e] Michael Ruck,[e, f] Ute Kaiser,[c] Inez M. Weidinger,[d] and Stefan Kaskel*[a,b]

Abstract: Clean air is an indispensable prerequisite for human health. The capture of small toxic molecules requires the development of advanced materials for air filtration. Two-dimensional nanomaterials offer highly accessible surface areas but for real-world applications their assembly into well-defined hierarchical mesostructures is essential. DUT-134(Cu) ($[Cu_2(dttc)_2]_n$, dttc = dithieno[3,2-b:2',3'-d]thiophene-2,6-dicarboxylate]) is a metal-organic framework forming platelet-shaped particles, that can be organized into complex structures, such as millimeter large free-standing layers (carpets) and tubes. The structured material demonstrates enhanced accessibility of open metal sites and significantly enhanced H_2S adsorption capacity in gas filtering tests compared with traditional bulk analogues.

DOI: 10.1002/anie.2022XXXXX

SUPPORTING INFORMATION

Table of Contents

Table of Contents 2
1. Chemicals and Methods 4
1.1 Chemicals 4
1.2 Instruments and Methods 4
1.2.1 Solution/liquid-state NMR 4
1.2.2 Mass spectrometry 5
1.2.3 Elemental analysis 5
1.2.4 X-ray diffraction 5
1.2.5 SEM and EDS measurement 5
1.2.6 Raman spectroscopy 5
1.2.7 Transmission Electron Microscopy (TEM) 5
1.2.8 Atomic force microscopy (AFM) 6
1.2.9 Physisorption 6
2. Synthesis of Organic Compounds 7
2.1 Synthetic approach for dithieno[3,2-b:2',3'-d]thiophene-2,6-dicarboxylic acid 7
2.1.1 3,4-Dibromo-2,5-diformylthiophene (b) 8
2.1.2 Dithieno[3,2-b:2',3'-d]thiophene-2,6-dicarboxylic acid diethyl ester (c) 8
2.1.3 Dithieno[3,2-b:2',3'-d]thiophene-2,6-dicarboxylic acid (d) 9
3. Synthesis DUT-134(Cu) 10
3.1 Synthesis of DUT-134·DMF bulk 10
3.2 Synthesis of DUT-134 bulk 10
3.3 Synthesis of DUT-134 carpets 10
4. Characterisation of DUT-134 11
4.1 Powder X-ray diffraction 11
4.2 Structural differences of solvated and desolvated DUT-134 12
4.3 Scanning electron microscopy of DUT-134 carpets 13
4.4 Energy dispersive X-ray analysis of DUT-134 carpets 14
4.5 SEM images of carpets deposited on surfaces 15
4.5.1 DUT-134 carpets on glass capillary 15
4.5.2 DUT-134 carpets deposited on flat substrates 15
4.6 Raman spectroscopy 16
4.6.1 DUT-134 bulk and DUT-134 carpets (desolvated) 16
4.6.2 DUT-134·DMF Raman studies: Bulk material and carpets in DMF and H_2O 17
4.7 Additional Information 18
4.8 H_2S adsorption 19
4.8.1 InfraSORP measurement 19

4.8.2 Breakthrough measurement 19

4.8.3 Powder X-ray diffraction patterns before and after H_2S treatment 21

4.8.4 Cyclisation of DUT-134 22

4.8.5 N_2 physisorption measurement at 77 K 23

4.8.6 SEM images of DUT-134 crystals before and after H_2S treatment 24

4.8.7 Raman spectroscopy after H_2S treatment 25

4.9 Atomic Force Microscopy (AFM) 26

References 27

1. Chemicals and Methods

1.1 Chemicals

All the reagents and solvents for synthesis were purchased from commercial sources and used as supplied without further purification.

Table S1. List of chemicals used for the synthesis of the ligand and MOF.

Name	CAS	purity	Manufacturer
**Acetone**	67-64-1	≥99.8%, extra dry	Fisher Scientific
**Copper(II) nitrate trihydrate**	10031-43-3	98%	Sigma Aldrich
**Dimethyl sulfoxide (DMSO)**	67-68-5	99.9%	Fisher Scientific
**Ethanol**	64-17-5	≥99.8	Sigma Aldrich
**Ethyl thioglycolate**	623-51-8	97%	Sigma Aldrich
**Hydrochloric acid**	7647-01-0	37%	Sigma Aldrich
**Lithium hydroxide**	1310-65-2	98%	Sigma Aldrich
***n*-Butyllithium**	109-72-8	2.5 M in hexanes	Sigma Aldrich
***N*-Formylpiperidine**	2591-86-8	99%	Sigma Aldrich
***N,N*-Dimethylformamide (DMF)**	109-72-8	99.5%	Fisher Scientific
***N,N*-Dimethylformamide (DMF)**	68-12-2	≥99.8%, extra dry	Fisher Scientific
**Potassium carbonate**	584-08-7	≥99%	Sigma Aldrich
**Tetrabromothiophene**	3958-03-0	>99%	TCI
**Tetrahydrofuran (THF)**	109-99-9	≥99%	Sigma Aldrich

1.2 Instruments and Methods

1.2.1 Solution/liquid-state NMR

Nuclear magnetic resonance (NMR) spectra were measured on a BRUKER DRX 500 P spectrometer (500.13/600.16 MHz and 125.77/150.91 MHz for ^{1}H and ^{13}C respectively) and/or on a BRUKER AC 300 P (300 MHz, 282 MHz and 75.5 MHz for ^{1}H and ^{13}C, respectively). All ^{1}H and ^{13}C NMR spectra are reported in parts per million (ppm) downfield of TMS and were measured relative to the residual signals of the solvents at 7.26 ppm ($CDCl_3$) and 2.50 ppm (DMSO). Data for ^{1}H NMR spectra are described as following: chemical shift (δ (ppm)), multiplicity

(s, singlet; t, triplet; q, quartet; br, broad signal), coupling constant J (Hz), integration corresponding to amount of H, C or CH. Data for ^{13}C NMR spectra are described in terms of chemical shift (δ (ppm)) and functionality were derived from DEPT spectra.

1.2.2 Mass spectrometry

High resolution-time of flight mass spectroscopy (HR-TOF-MS) was carried out on a maXis TOF of Bruker. The samples were dissolved in dimethyl sulfoxide or ethyl acetate and diluted with a solution of 1% ammonium formate in methanol. The solution was ionized by electron spray ionisation.

1.2.3 Elemental analysis

Elemental analysis experiments were performed on a HEKAtech EA 3000 Euro Vector CHNSO elementaranalysator in CHNS modus. The composition was determined as the average of three individual measurements on three individually prepared samples. Guest free powder of DUT-134 was analysed by placing 3 - 5 mg of sample in tin sample holders under inert atmosphere in a glove box and sealed to avoid contamination by humidity.

1.2.4 X-ray diffraction

Powder X-ray diffraction (PXRD) experiments at room temperature were carried out on a STOE STADI P diffractometer with Cu Kα1 radiation (λ = 1.5405 Å) and a 2D detector (Mythen, Dectris) in transmission geometry. A scan speed of 120 s/step and a detector step size of 3° was used in the measurements. The samples for X-ray diffraction analysis were placed between non-diffracting sheets of a MPET-foil (rescue blanket). Activated samples were prepared under inert atmosphere (in an Ar filled glove box). PXRD patterns were calculated from single crystal structure data using Mercury 3.9 software package.

1.2.5 SEM and EDS measurement

Scanning electron microscopy (SEM) measurements were carried out using a 2 kV acceleration voltage and a working distance of 12 mm on a SU8020 from Hitachi. Prior to the measurements, the samples were sputtered with Au to increase the conductivity. Energy-dispersive X-ray spectroscopy (EDS) measurements were made on the same instrument. The sample was dripped from suspension onto a silicon wafer, which was fixed on the sample holder by a carbon pad. The measurements were performed with a voltage of 20 kV and a current between 15 and 20 µA with a working distance of 15 mm. To prevent overlapping X-ray emission peaks of gold and sulphur, the samples were not sputtered for this type of measurement.

1.2.6 Raman spectroscopy

Raman measurements were performed with the Confocal Raman Microscope (CRS+) MonoVista from Spectroscopy & Imaging GmbH using lasers with wavelengths of 514 nm and 785 nm. Supercritically dried samples were measured under inert conditions (Ar atmosphere) in quartz glass fluorescence cuvettes (1 cm path length).

1.2.7 Transmission Electron Microscopy (TEM)

For TEM investigations an amorphous-carbon coated copper TEM grid was placed into a vial containing dry carpet sample. The vial was sealed and agitated in order to facilitate adhesion of the carpets to the grid. The carpets were easily recognizable on the grid due to their large lateral dimensions.

The TEM measurements were performed using a Thermofischer TITAN TEM operating at 300 kV. We used relatively low electron dose rate of 5e/Å^{2}s, in order to minimise damage of the lattice by the electron beam. Further transmission electron microscopy images were performed on a JEM-1400 Plus device from JOEL with a voltage of 120 kV (LaB_6 cathode). Small amounts of sample were suspended in ethanol and dropped (10 µL) onto carbon coated Cu mesh. The respective magnifications are shown in the images.

1.2.8 Atomic force microscopy (AFM)

The layer thickness was measured in tapping mode at a measuring speed of 2 - 5 µm/s using the Dimension D 3000 atomic force microscope (AFM) from Digital Instruments (Bruker).

1.2.9 Physisorption

Physisorption measurements were performed on Autosorb IQ (for N_2) and on BELSORP MAX II apparatus (for Kr). The dead volumes of the measuring system and the sample cell were routinely determined using helium. The adsorption of Kr was investigated on the desolvated DUT-134_carpet samples. To ensure complete removal of the solvent molecules (especially from open metal sites of the Cu paddle wheel cluster), the samples were treated in the dynamic vacuum of 10^{-1} Pa at 383 K for at least 24 h prior to the measurements.

2. Synthesis of Organic Compounds

2.1 Synthetic approach for dithieno[3,2-b:2',3'-d]thiophene-2,6-dicarboxylic acid

For the synthesis of the linker dithieno[3,2-b:2',3'-d]thiophene-2,6-dicarboxylic acid (H_2dttc), a previously described [1] but slightly optimised approach was used.

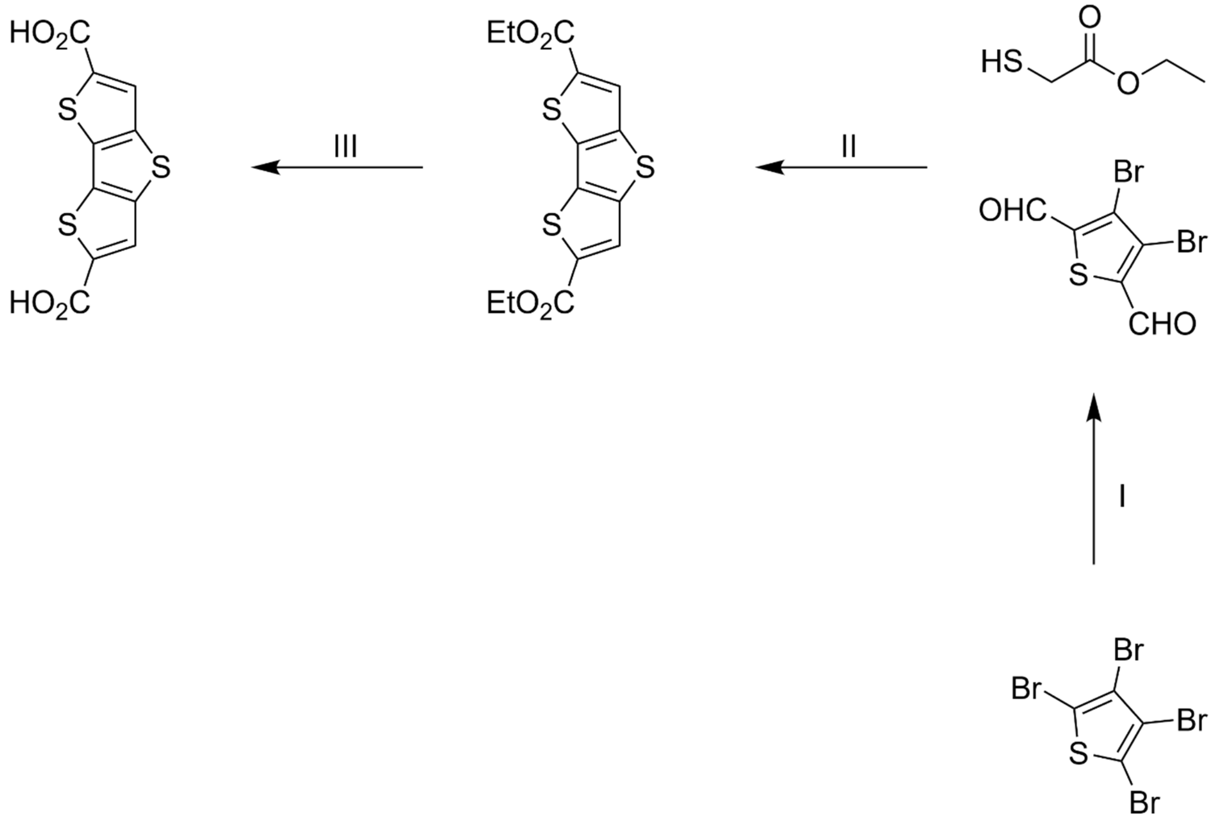

Figure S1. Three step synthetic approach for H_2dttc: (**I**) *n*-BuLi, *N*-formylpiperidine, THF; (**II**) K_2CO_3, DMF; (**III**) LiOH, H_2O, THF.

2.1.1 3,4-Dibromo-2,5-diformylthiophene (b)

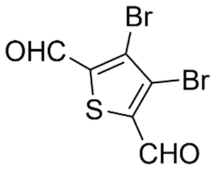

A 1 L flame dried round bottom Schlenk flask was charged with 35 g (87.6 mmol, 1.00 eq.) tetrabromothiophene. The flask was evacuated and flushed with argon three times before dissolving the solid in 400 mL degassed anhydrous THF. After the solution was cooled to -78 °C with a mixture of dry ice and acetone and stirred for 10 min, 75.3 mL (188.3 mmol, 2.5 M, 2.15 eq.) *n*-BuLi was added dropwise over a period of one hour. After addition, the brown solution was stirred in an ice bath for approx. 15 min at 0 °C to ensure complete lithiation. The reaction mixture was then cooled again to -78 °C and 21.86 mL (197.0 mmol, 2.25 eq.) N-formylpiperidine was added quickly. The solution was then allowed to slowly warm up to room temperature overnight. The reaction mixture was cooled by an ice bath before 200 mL (6 M) hydrochloric acid was added to obtain a yellowish precipitate. The suspension was stirred for another 60 min at 0 °C, then filtered immediately through a sintered-glass funnel. The resulting solid was washed with water and a small amount of EtOH before being dried in a drying oven at 60 °C for 24 h to obtain 23.7 g (91%) of the crude dialdehyde (b). The material was used without further purification steps as a starting material for further syntheses.

^{1}H NMR (500 MHz, $CDCl_3$) δ (ppm): 10.07 (s, 2 H).

^{13}C NMR (151 MHz, $CDCl_3$) δ (ppm): 123.7 (s, 1 C), 142.3 (s, 1 C), 183.3 (s, 1 CH).

2.1.2 Dithieno[3,2-b:2',3'-d]thiophene-2,6-dicarboxylic acid diethyl ester (c)

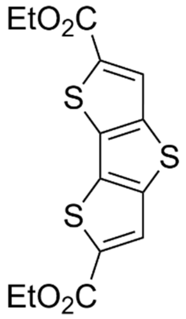

A 1 L flame dried round bottom Schlenk flask was charged with 16 g (53.7 mmol, 1.00 eq.) 3,4-dibromo-2,5-diformylthiophene (b). The flask was evacuated and flushed with argon three times before dissolving the solid in 500 mL degassed anhydrous DMF. 20 g (161.1 mmol, 3.00 eq.) of anhydrous potassium carbonate was added to the reaction mixture, resulting in a dark green, almost black suspension and 12.4 mL (112.8 mmol, 2.10 eq.) Ethyl 2-mercaptoacetate was then added dropwise over a period of about 15 min. The mixture was stirred for 72 h under Ar atmosphere at room temperature. The resulting reddish brown suspension was transferred to a beaker containing 600 mL of water and a yellow residue precipitated, which was filtered, washed with water and little EtOH. The separated aqueous phase (filtrate of the precipitate) was extracted 3 times with ca. 250 ml of dichloromethane. The organic phases were collected, twice washed with 200 mL brine, dried over $MgSO_4$ and filtered through a sintered-glass funnel. Excess solvent was evaporated on the rotary evaporator up to a volume of approx. 100 mL whereby the first crystallisation could already be observed. At low temperatures (4°C refrigerator), a lot of solid material formed in the form of yellow crystals, which were also filtered and treated as described in the purification procedure. The washed and combined product of 15.9 g (87%) dithieno[3,2-b:2',3'-d]thiophene-2,6-dicarboxylic acid diethyl ester (c) could be obtained after drying for 24h in an 80 °C oven. The material was used without further purification steps as a starting material for further syntheses.

^{1}H NMR (500 MHz, $CDCl_3$) δ (ppm): 1.42 (t, J = 7.5 Hz, 4 H), 4.40 (q, J = 7.5 Hz, 6 H), 8.00 (s, 2 H).

^{13}C NMR (151 MHz, $CDCl_3$) δ (ppm): 14.47 (s, 1 CH_3), 61.84 (s, 1 CH_2), 126.84 (s, 1 CH), 135.35 (s, 1 C), 135.98 (s, 1 C), 143.40 (s, 1 C), 162.21 (s, 1 C).

2.1.3 Dithieno[3,2-b:2',3'-d]thiophene-2,6-dicarboxylic acid (d)

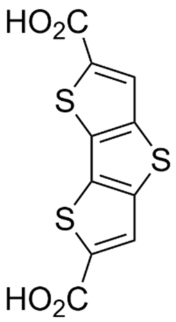

In a 250 ml round bottom flask 5 g (14.7 mmol, 1.00 eq.) dithieno[3,2-b:2',3'-d]thiophene-2,6-dicarboxylic acid diethyl ester (c) was dissolved in 175 mL THF, mixed with 175 mL (1 M, 12.00 eq.) Lithium hydroxide solution and refluxed for 4 h, while a colour of the suspension changes from yellow to orange and already a small amount of precipitate could be observed. After the reaction, THF was removed by rotary evaporator and a white solid was precipitated by an excess of HCl (6 M). The white solid was filtered, washed with water and small amount of EtOH, and dried overnight in a vacuum oven (60 °C, 100 Pa) to obtain 3.97 g (95%) of the crude dicarboxylic acid (d).

^{1}H NMR (500 MHz, DMSO-d_6) δ (ppm): 8.22 (s, 2 H), 13.49 (s, 2 H).

^{13}C NMR (151 MHz, DMSO-d_6) δ (ppm): 127.60 (s, 1 CH), 134.15 (s, 1 C), 136.60 (s, 1 C), 143.61 (s, 1 C), 163.06 (s, 1 C).

(ESI (-)) (m/z): 283 [M-H], 567 [2M-H].

3. Synthesis DUT-134(Cu)

3.1 Synthesis of DUT-134·DMF bulk

The synthesis was carried out according to the previously published method.[2] 43.5 mg (0.15 mmol, 1.0 eq.) of dithieno[3,2-b:2',3'-d]thiophene-2,6-dicarboxylic acid (H_2dttc) was dissolved in 6.7 mL dimethyl sulfoxide (DMSO) using ultrasound and placed in a 25 mL autoclave vessel. On the top of the ligand solution, a mixture of 3.35 mL DMSO/EtOH (1:1) was placed. After that, a solution of 67.0 mg $Cu(NO_3)_2 \cdot 3H_2O$ (0.28 mmol, 1.8 eq.) in 6.7 mL ethanol was added as an upper layer. The autoclave was heated at 60 °C for 3 d and at 90 °C for 5 d to obtain DUT-34(Cu)·DMF ($[Cu_2(dttc)_2(DMF)_2(L)_x]_n$) with a yield of 95%. After cooling down the reaction mixture, the obtained dark green crystals were washed with DMF three times. The phase purity was confirmed by powder X-ray diffraction (Fig. S2).

3.2 Synthesis of DUT-134 bulk

To remove DMF from the pores and especially from the free coordination sites of the Cu paddle wheel, a solvent exchange to anhydrous acetone was carried out first for at least two times per day over a period of 5 d. Afterwards the material was desolvated by means of supercritical CO_2 drying (SCD). The obtained solvent-free, dark green crystals were handled in an argon-filled glove box.

^{1}H NMR (300 MHz, DMSO-d_6/DCl) δ (ppm): 8.21 (s, 2 H).

Elemental analysis: Calculated ($Cu_2(dttc)_2$): C: 34.73%; H: 0.58%; N: 0%; S: 27.81%; found: C: 34.61%; H: 0.57%; N: 0%; S: 27.71%.

3.3 Synthesis of DUT-134 carpets

The quasi-interfacial synthesis approach[3] was used to synthesize platelets seeds of DUT-134(Cu)·DMF first. For that, H_2dttc (21 mg, 0.07 mmol, 1.0 eq.) was dissolved in a 2:1 mixture of DMSO and DMF (2 ml and 1 ml, respectively) via ultrasonication and placed in a glass tube with 16 mm in diameter. As a buffer layer, a solvent mixture of 1 mL DMSO and 1 mL DMF was carefully added on top of the linker solution. The buffer layer was covered by the metal precursor solution obtained from sonication of $Cu(NO_3)_2 \cdot 3H_2O$ (32 mg, 0.13 mmol, 1.8 eq.) in a mixture of 1 mL DMSO and 2 mL DMF. The starting materials ratio was taken from the synthesis of DUT-134·DMF[2] and scaled down to the amount of solvent used.

The glass tube was then sealed with a screw cap and heated to 60 °C for 5 d. Subsequently, after centrifugation of the cooled solutions at 1500 rpm for 10 min, a small amount of white precipitate was obtained. The supernatant solution was carefully removed and replaced by fresh DMF. Then, the precipitate was dispersed in DMF by shaking. Afterward, the tube was allowed to rest for 4 d at RT.

The obtained semi-transparent MOF "carpets" were washed 3 times with DMF to remove precursor residues. For physisorption measurements, TEM, and SEM analyses, $[Cu_2(dttc)_2]_n$ carpets were dried supercritically using CO_2 after solvent exchange to acetone over several days.

4. Characterisation of DUT-134

4.1 Powder X-ray diffraction

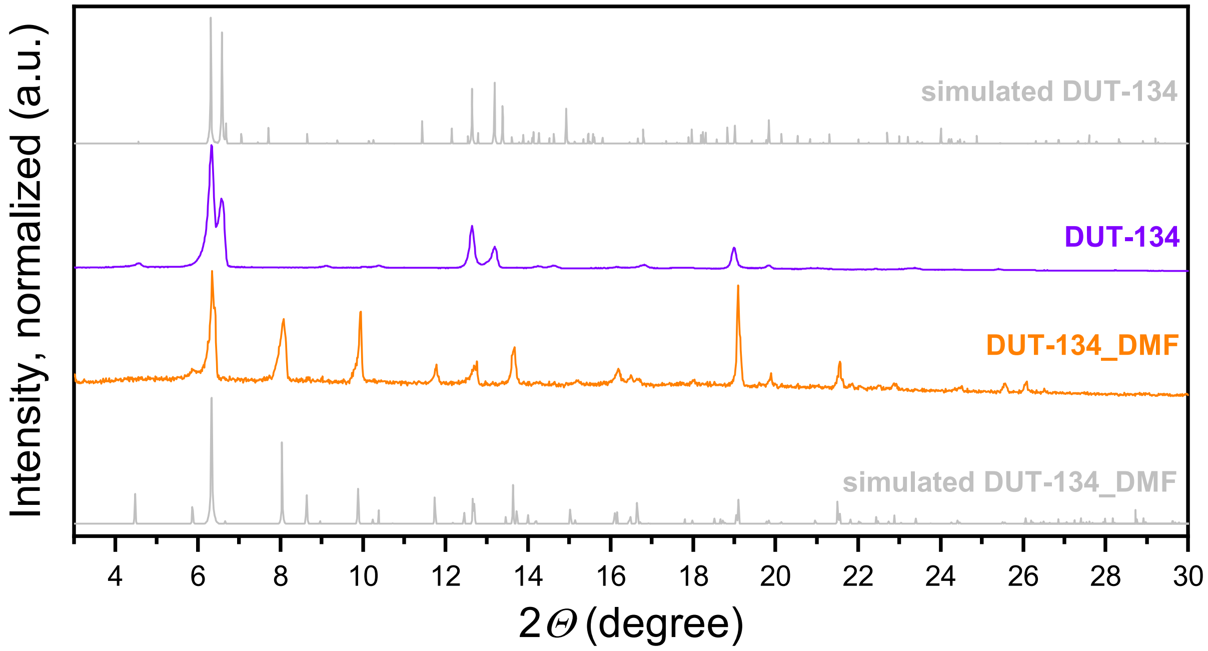

Figure S2. X-ray powder diffraction pattern of DUT-134 bulk crystals and corresponding simulated patterns.

4.2 Structural differences of solvated and desolvated DUT-134

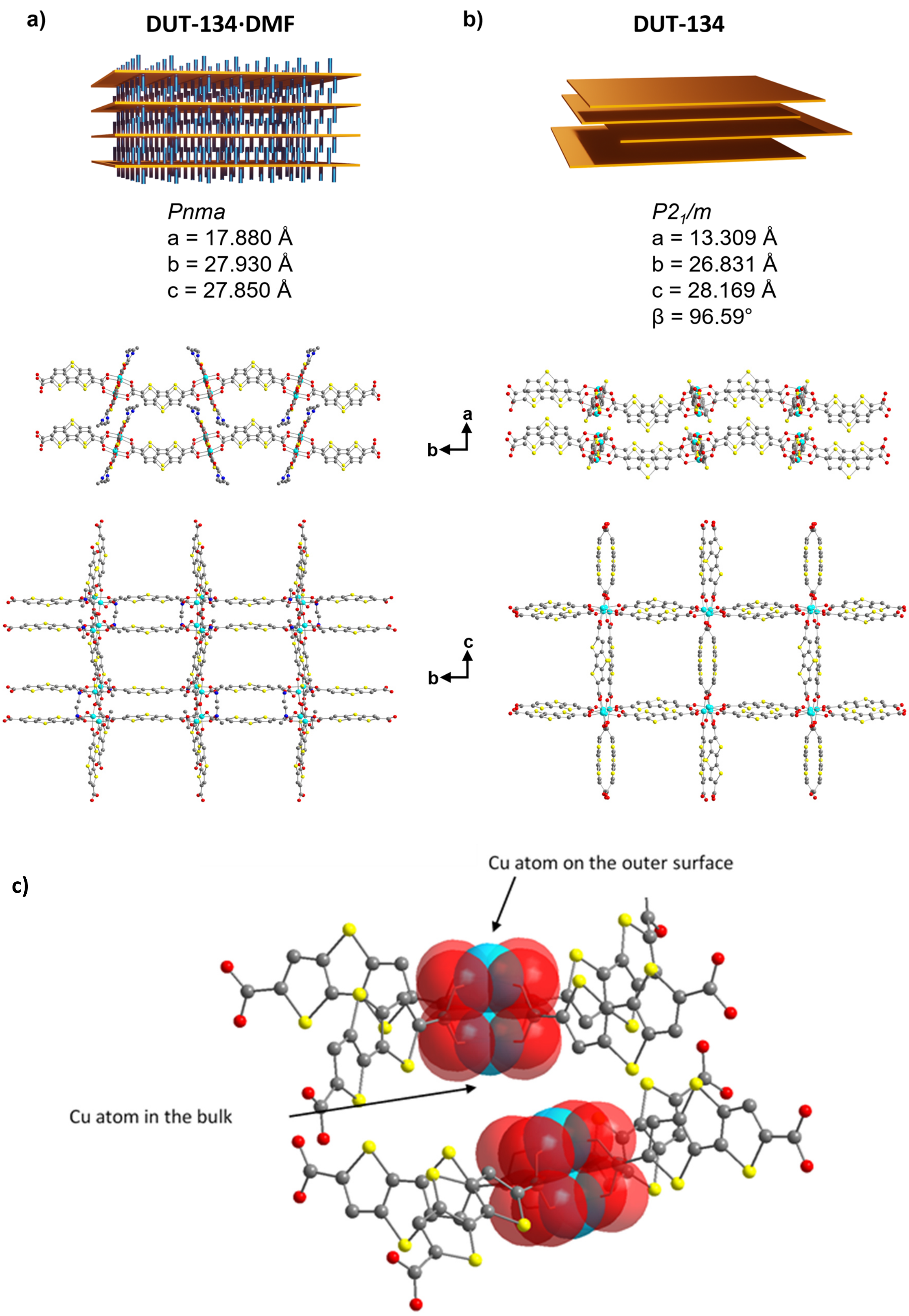

Figure S3. Schematic illustration, unit cell parameters and view along the *c* and *a* axes on the DMF solvated (a) and solvent-free forms (b) of DUT-134; c) The environment of the Cu atoms in desolvated bulk material (inner surface) and on the external surface.

4.3 Scanning electron microscopy of DUT-134 carpets

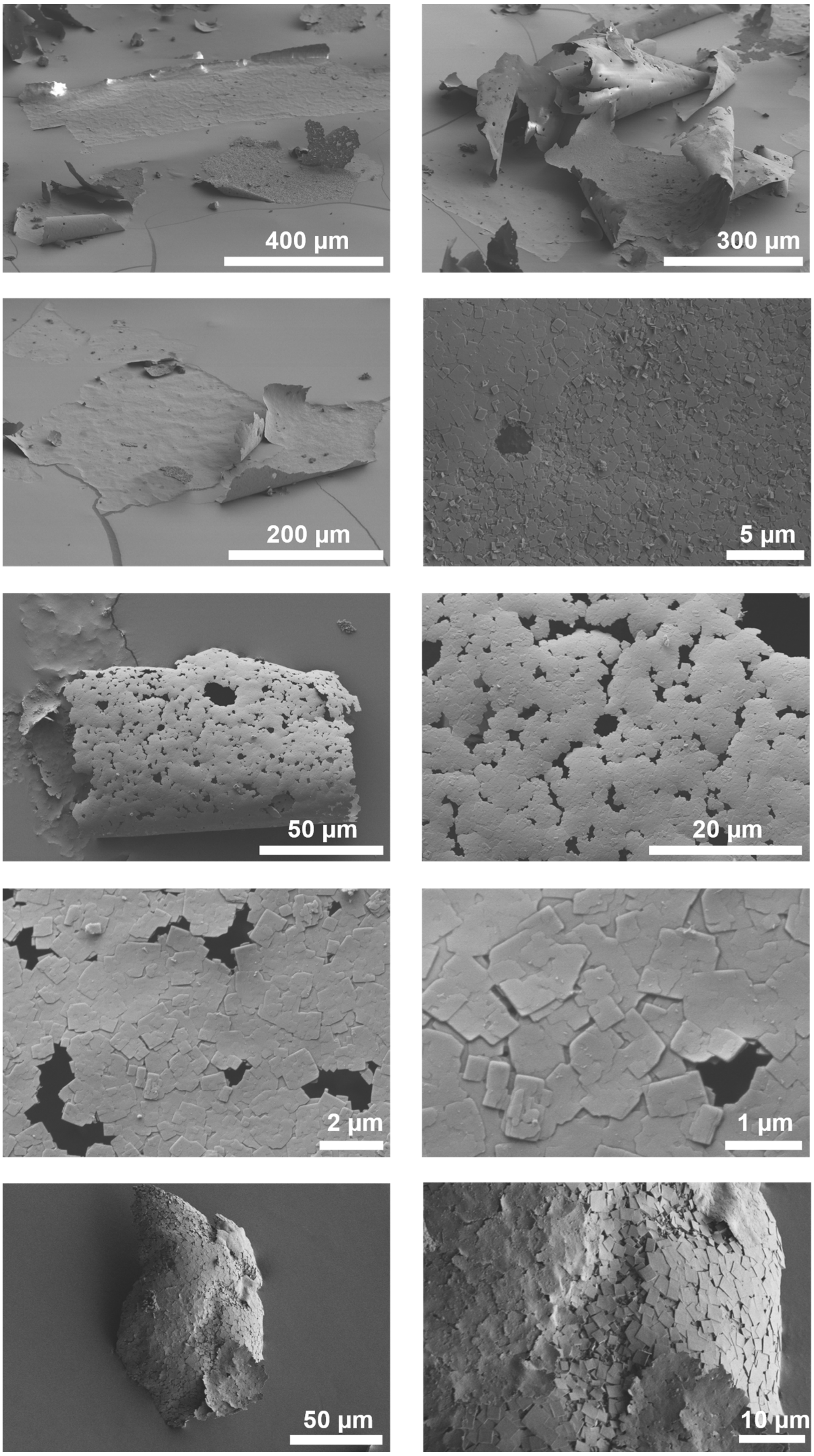

Figure S4. SEM images of DUT-134 carpets.

4.4 Energy dispersive X-ray analysis of DUT-134 carpets

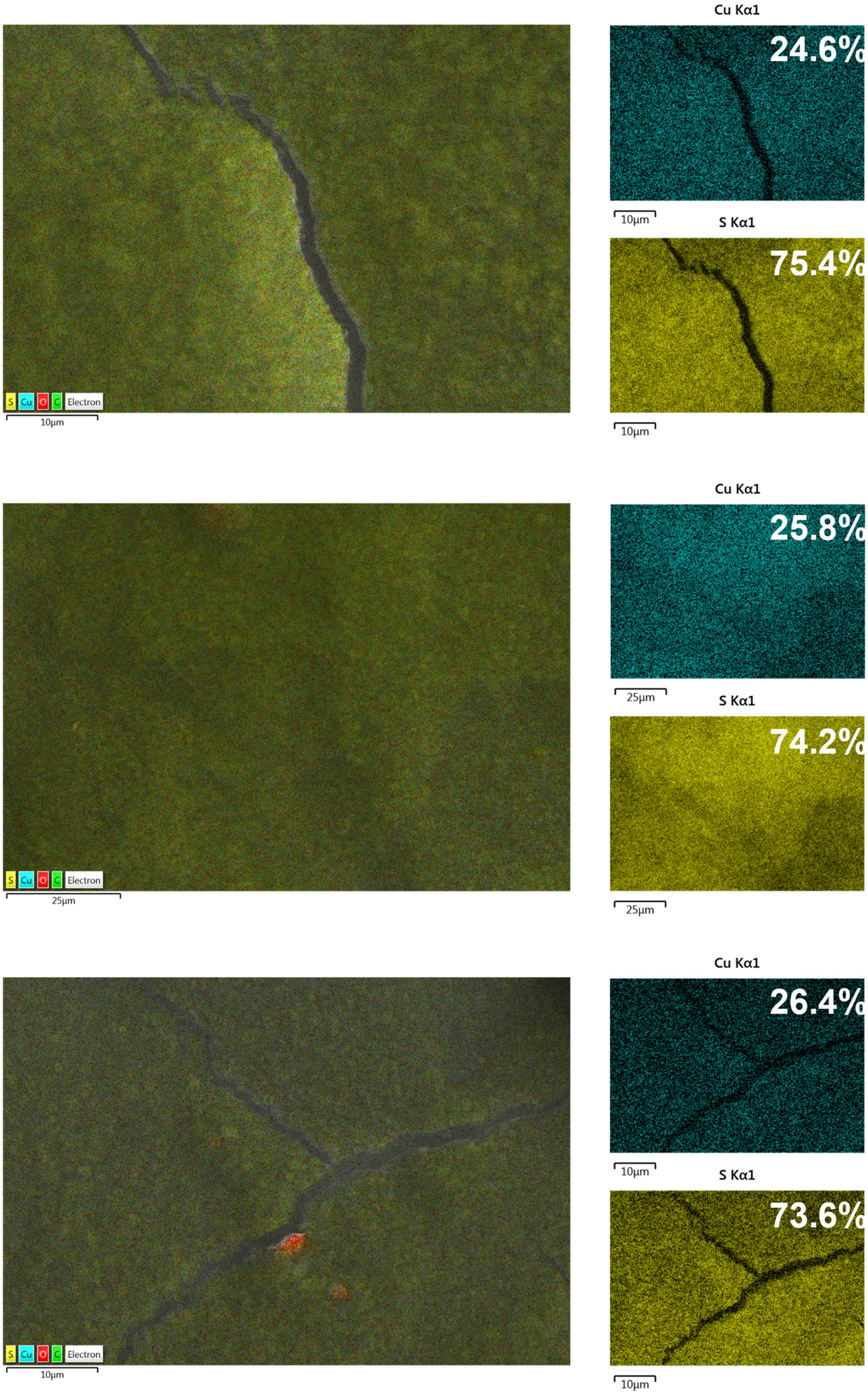

Figure S5. EDX mapping on DUT-134(Cu) carpets. S: yellow, Cu: blue, O: red, C: green. The molecular formula $[Cu_2(dttc)_2]_n$ is expected for supercritically dried MOF crystals. The atomic ratio of copper and sulfur in the coordination compound is accordingly 1:3, which is in excellent agreement with the EDX measurements.

4.5 SEM images of carpets deposited on surfaces

4.5.1 DUT-134 carpets on glass capillary

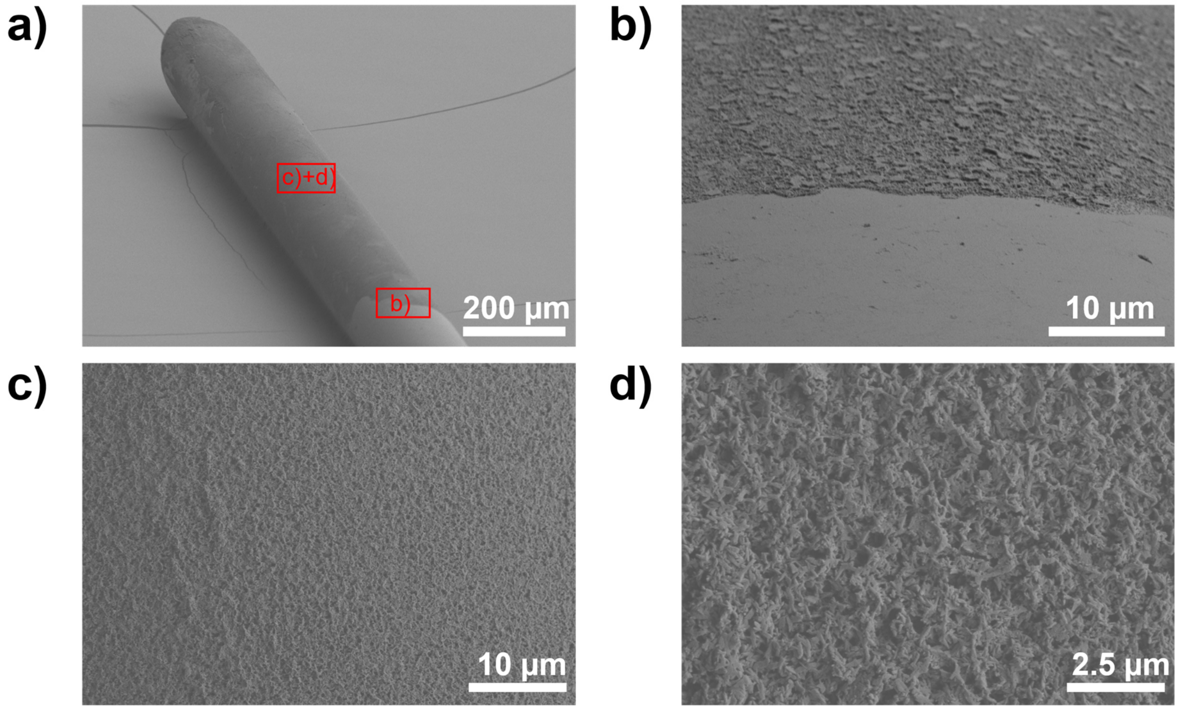

Figure S6. SEM images of DUT-134 carpets deposited on the glass capillary.

4.5.2 DUT-134 carpets deposited on flat substrates

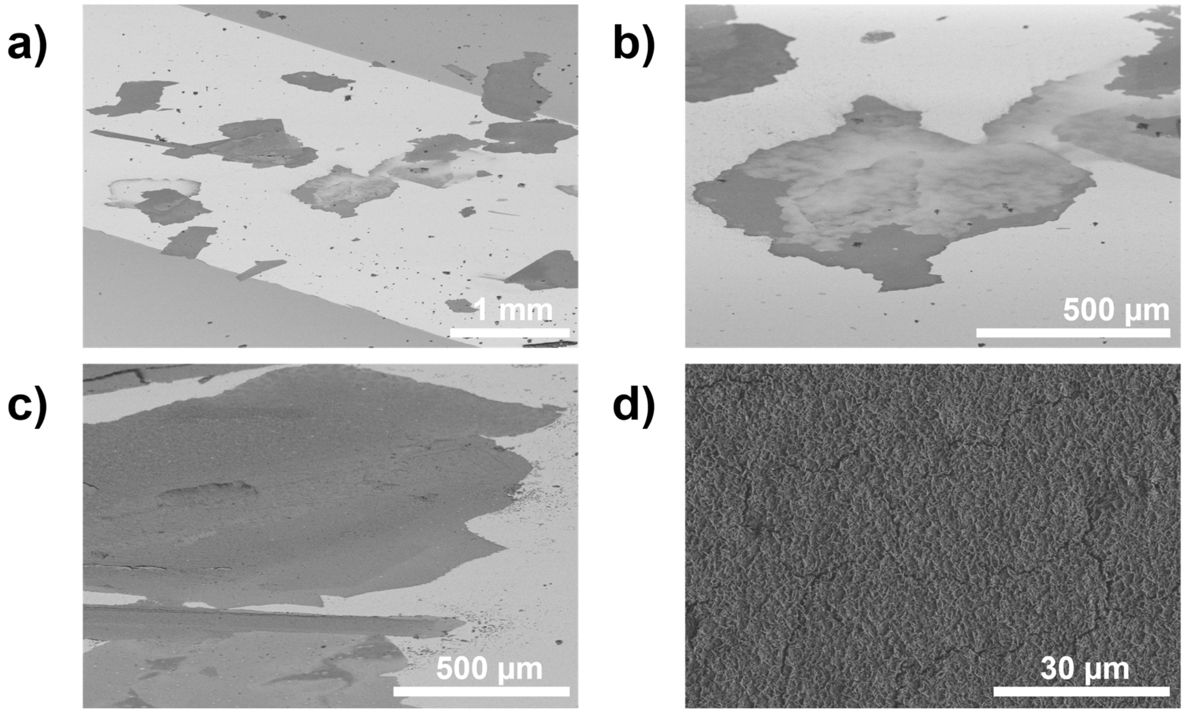

Figure S7. SEM images of DUT-134 carpets deposited on: a) and b) Au surface; c) and d) Si-wafer.

4.6 Raman spectroscopy

4.6.1 DUT-134 bulk and DUT-134 carpets (desolvated)

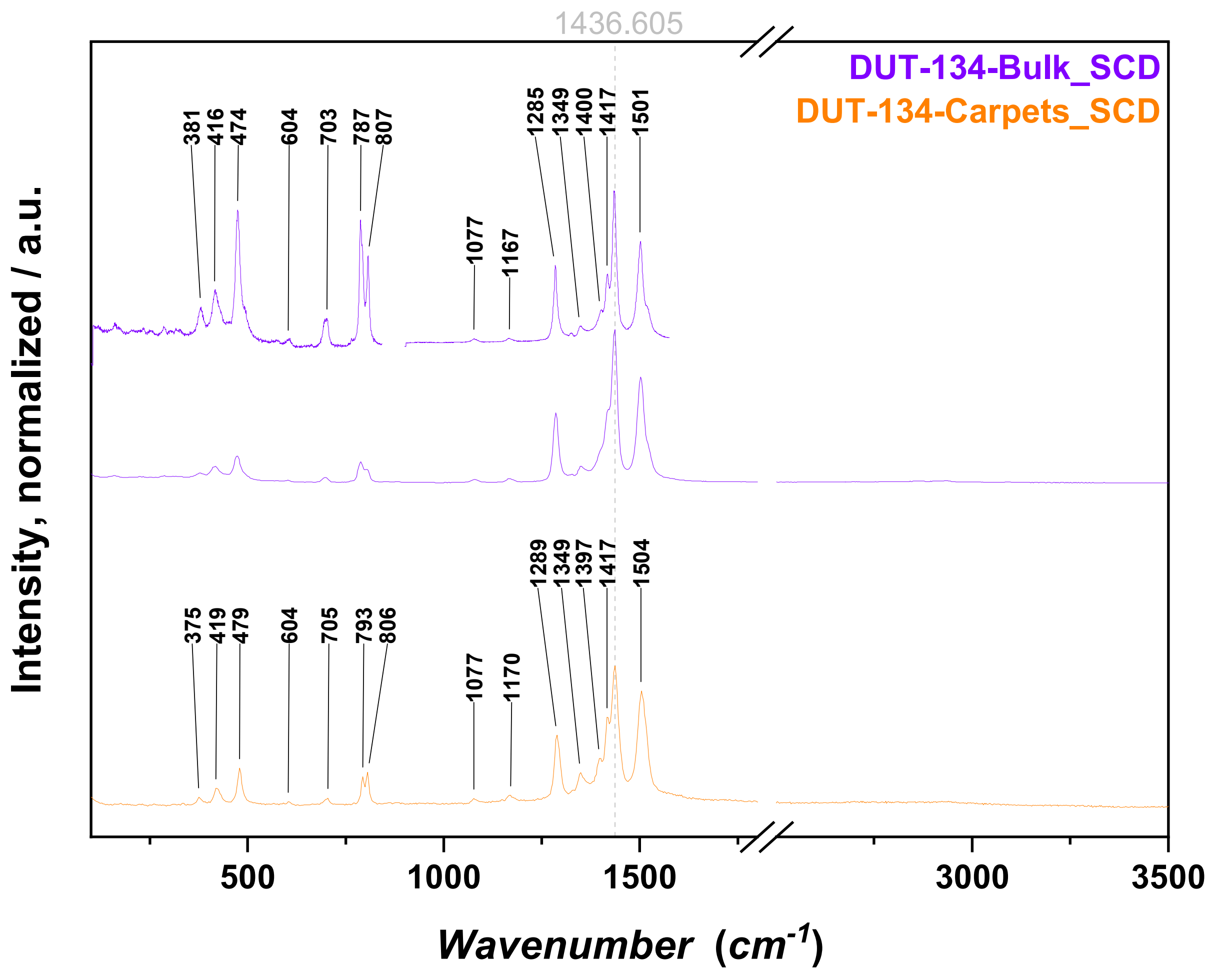

Figure S8. Vibrational spectra of DUT-134.

4.6.2 *DUT-134·DMF Raman studies: Bulk material and carpets in DMF and* H_2O*.*

From these data, it can be clearly seen that DUT-134 is stable in both DMF and water. In particular, however, it must be mentioned that the carpets have a lower signal-to-noise ratio due to the smaller layer thickness of approximately 200 nm, which can result in interference with the vibrations of solvent molecules (*Fig. S9*a). Water is only very weakly Raman-active, which makes the identification of the vibrational spectrum much easier (Fig. S9b). However, in both cases, the specific motif of the DUT-134 vibrational spectrum can be identified.

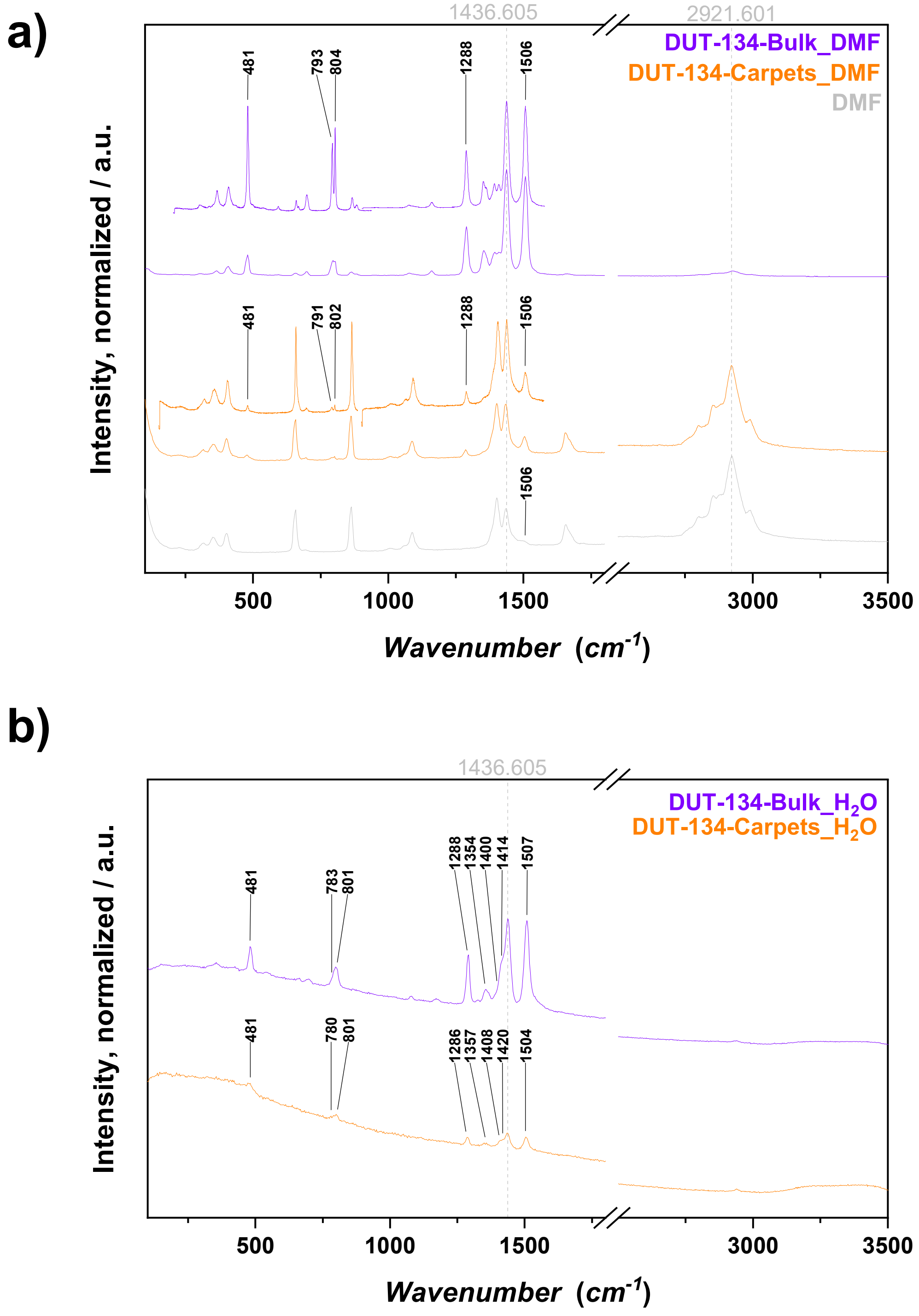

Figure S9. Raman spectra of DUT-134·DMF bulk and carpets: (a) after 7 d in DMF; (b) after 7 d in water.

4.7 Additional Information

Layered MOFs are expected to have lower packing density in comparison to their 3D counterparts[4], which is clearly seen in following image:

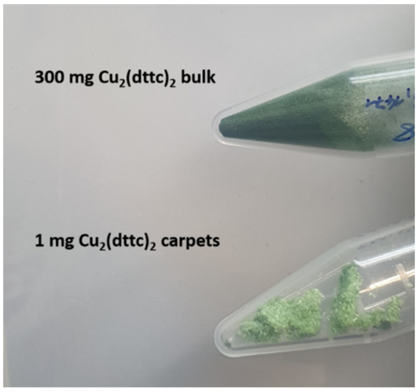

Figure S10. Packing density of carpets in comparison to the bulk material.

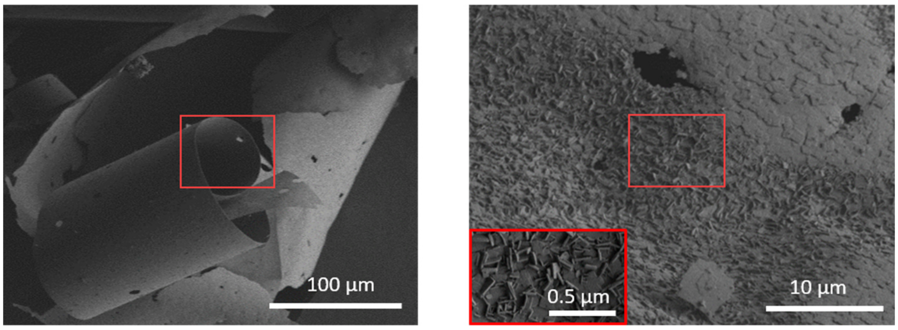

Figure S11. SEM images of carpets.

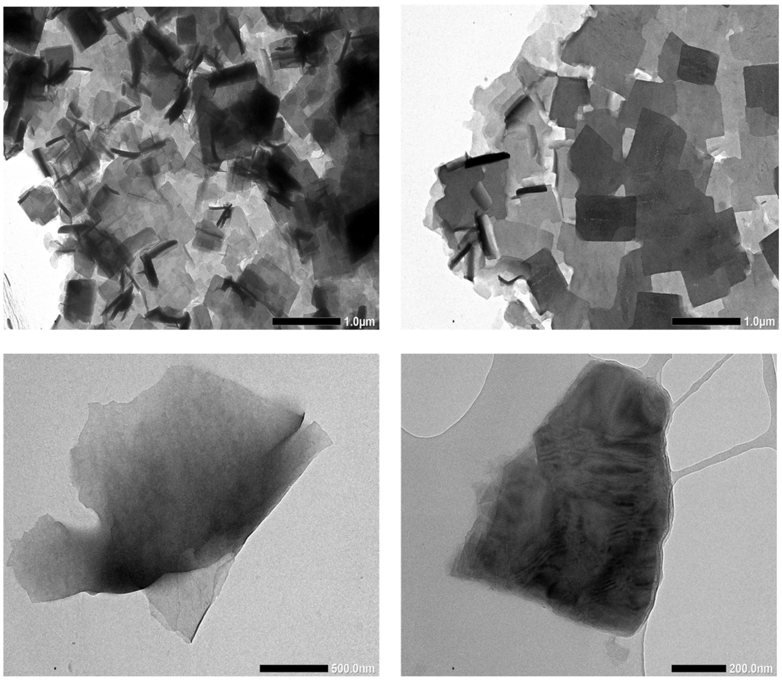

Figure S12. TEM images of carpets at varying magnifications.

4.8 H_2S adsorption

Adsorption is an exothermic process in which the heat of adsorption is released. This process can be described in terms of a reaction:

Adsorptive + Adsorbent ⇄ Adsorbate + Adsorption heat (ΔH_{ads})

InfraSORP technology is an extremely fast and efficient measuring tool for determining the H_2S adsorption capacity by detection of ΔH_{ads}. An infrared detector is used to detect the temperature change due to the heat release during gas adsorption process. If the temperature response signal is plotted against time and integrated, the uptake capacity of the sample can be determined (The peak area was calculated here using the Origin 20 software).

Subsequently, this area value was divided by the sample mass to obtain the InfraSORP activity in any comparable unit (K*s*mg^{-1}).[5]

For the measurements in this study, the material was placed in a sample holder under Ar atmosphere. After installing the sample in the instrument, the sample was purged for 10 min under a 70 mL/min flow of nitrogen (N_2, 99.999%) as an inert gas. Under the same flow, this gas was replaced by hydrogen sulfide (H_2S, 5000 ppm in N_2), which was flushed through the adsorbent, generating heat from the adsorption reaction of the gas with the material. The thermal response over time was detected by an infrared sensor. The sample was left under the H_2S flow until temperature reached the initial value.

4.8.1 InfraSORP measurement

Due to the low mass of the carpets of only 0.2 mg (HKUST-1: 35.60 mg; DUT-134 bulk: 31.83 mg), this measurement should be evaluated with great caution. Nevertheless, the trend already observed in the H_2S breakthrough measurement can be confirmed. Due to the 2D structure of carpets, diffusion limitation can be prevented to a large extent. This is also evident from the kinetics of adsorption process.

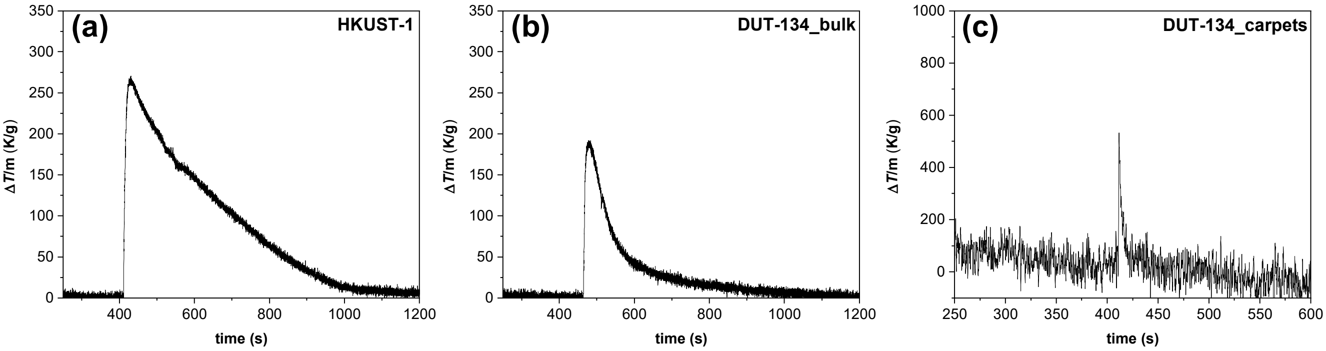

Figure S13. Temperature change in the InfraSORP cell: (a) HKUST-1; (b) DUT-134 bulk; (c) DUT-134 carpets.

4.8.2 Breakthrough measurement

Prior to all breakthrough experiments the sample was mixed with inert sand (mesh: 100 to 300 µm) resulting in a 1:3 mixture to reduce the measurement dead volume. A cylindrical stainless-steel cell (7.2 mm in diameter) was used as the adsorption column. This sample in the column was flushed with N_2 first. All the measurements were performed at room temperature, under a gas flow of 380 mL H_2S (1000 ppm in N_2) per minute. The amount of gas was controlled by a mass flow controller. An electrochemical sensor H_2S-BE (Alphasense) was used to record the H_2S concentration. As soon as the concentration reached its initial value, the measurement was stopped and the sample was purged with inert gas (Fig. S14). The breakthrough capacity was calculated using the integrated area above the breakthrough curve, the mass of sample, and the flow rate.

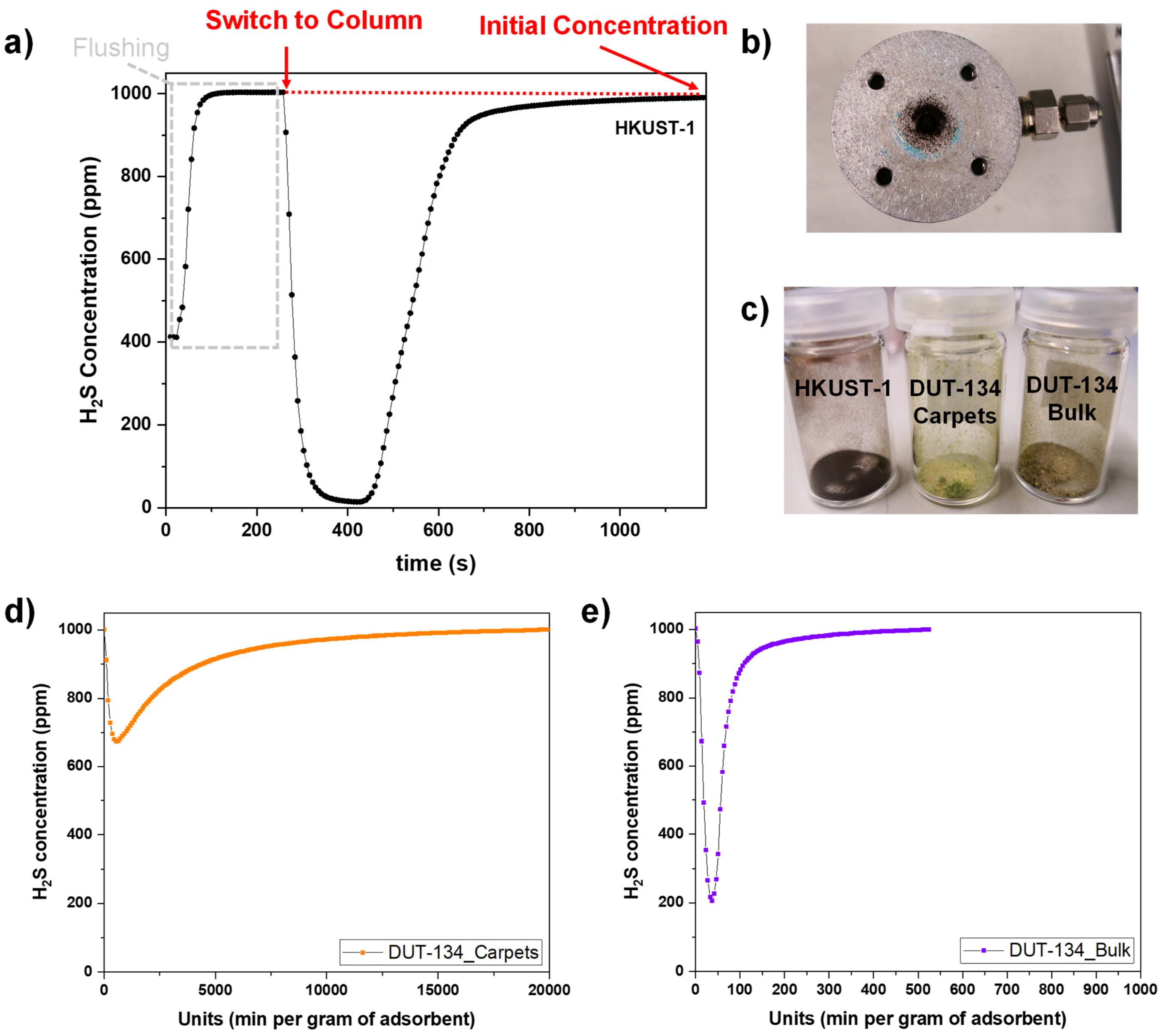

Figure S14. a) Typical breakthrough curve of HKUST-1; b) Column after H_2S treatment, color change from purple (HKUST-1, activated for 24 h at 150°C) to black (chemisorption); c) image of HKUST-1, DUT-134_carpets and DUT-134 after H_2S treatment; d) Breakthrough curve of DUT-134 carpets; e) Breakthrough curve of DUT-134 polycrystalline powder.

4.8.3 Powder X-ray diffraction patterns before and after H_2S treatment

The treatment of HKUST-1 with H_2S leads to an amorphization of the structure. The peak at 2θ = 22.92 is already present before the H_2S experiments, this contamination has no influence on the experiments (Fig. S15a). If DUT-134 comes into contact with H_2S, only a partial amorphization of the structure occurs (Fig. S15b). The signal-to-noise ratio increases, but the coordination network remains mostly intact. In the solvent-free form of DUT-134, the free copper coordination centers are embedded in the pore walls and are shielded from H_2S. Nevertheless, the material has impressive capacities due to its enormous surface. Additional reflections present in the diffraction pattern of the materials after the H_2S measurements belong to SiO_2, which was used as inert filler in the column.

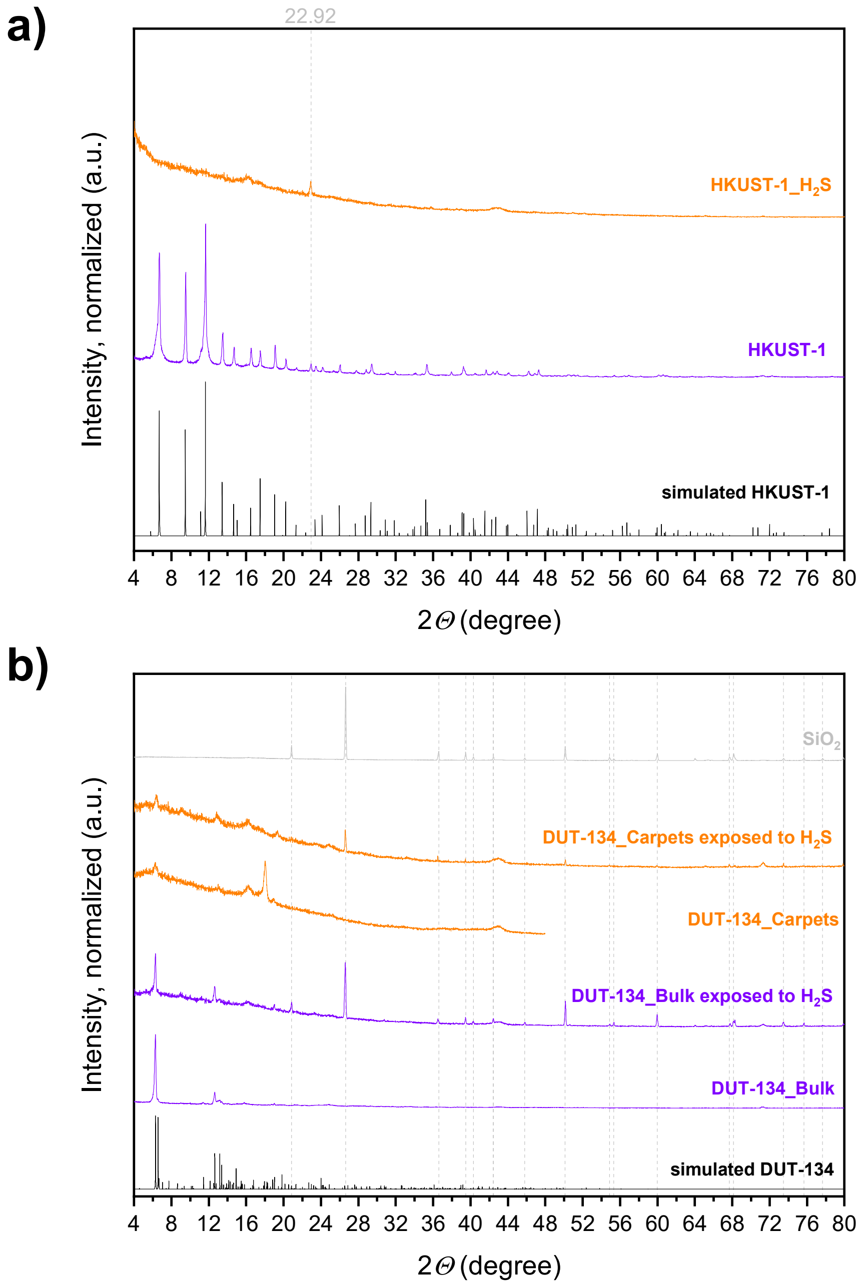

Figure S15. Powder X-ray diffraction patterns before and after H_2S treatment: a) reference material HKUST-1; b) DUT-134 bulk and DUT-134 carpets.

4.8.4 Cyclisation of DUT-134

To study the recyclability of the material, cyclic H_2S breakthrough experiments were carried out. As it can be seen from the figure below, the capacity in the first cycle is higher (86 mg/g) and drops in the second cycle to 26 mg/g and remains almost the same in the third cycle. It is reasonable to assume that in the first cycle chemisorption on Cu centers takes places, which is irreversible and in the next cycles only the physisorption is responsible for the uptake. The chemisorbed amount is 0.6 mol H_2S per 1 mol Cu.

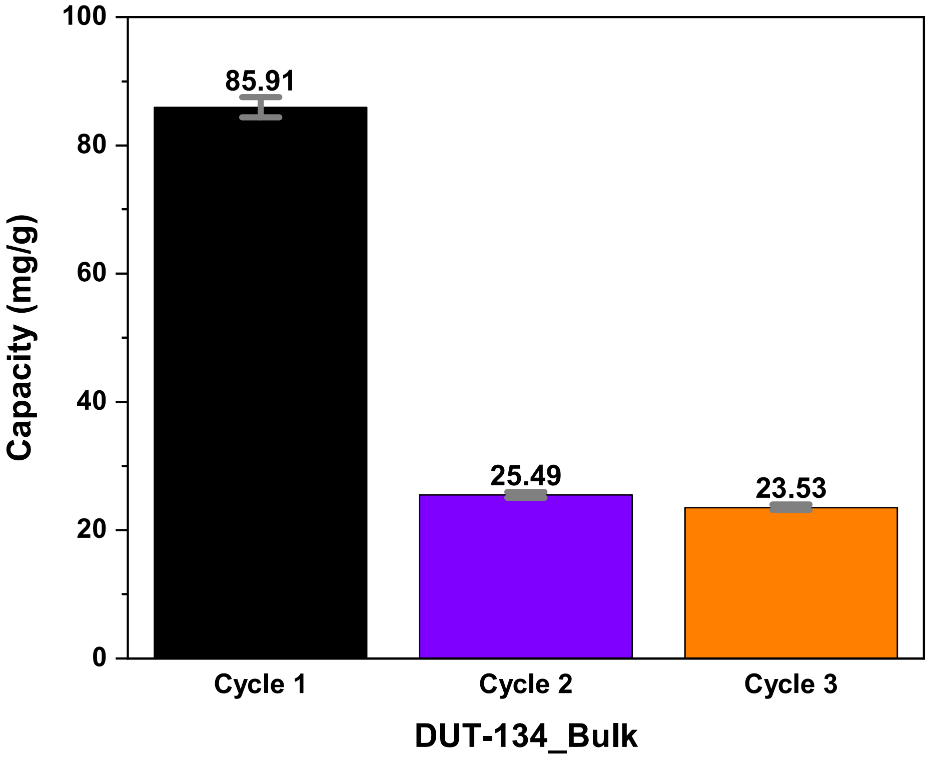

Figure S16. Capacity of DUT-134 after different H_2S treatments.

4.8.5 N_2 physisorption measurement at 77 K

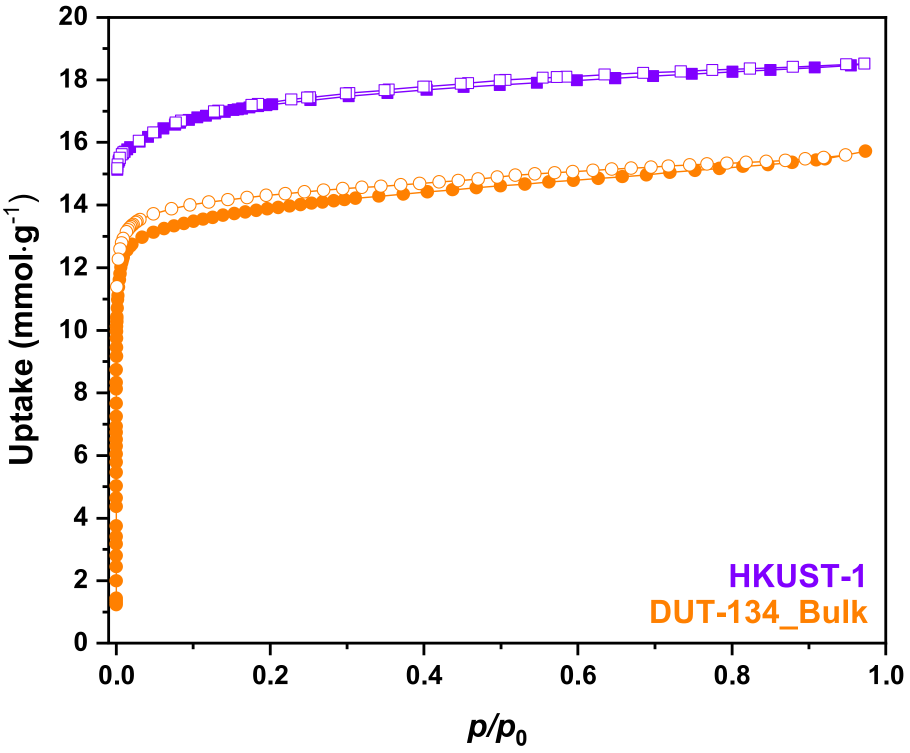

Figure S17. N_2 physisorption isotherm at 77 K of reference material HKUST-1 (purple; activation before measurement: 150 °C for 72 h; BET surface area: 1521 m^2/g) and DUT-134 bulk (orange; activation before measurement: 120 °C for 72 h; BET surface area: 1234 m^2/g). Solid symbols – adsorption, open symbols – desorption.

4.8.6 SEM images of DUT-134 crystals before and after H_2S treatment

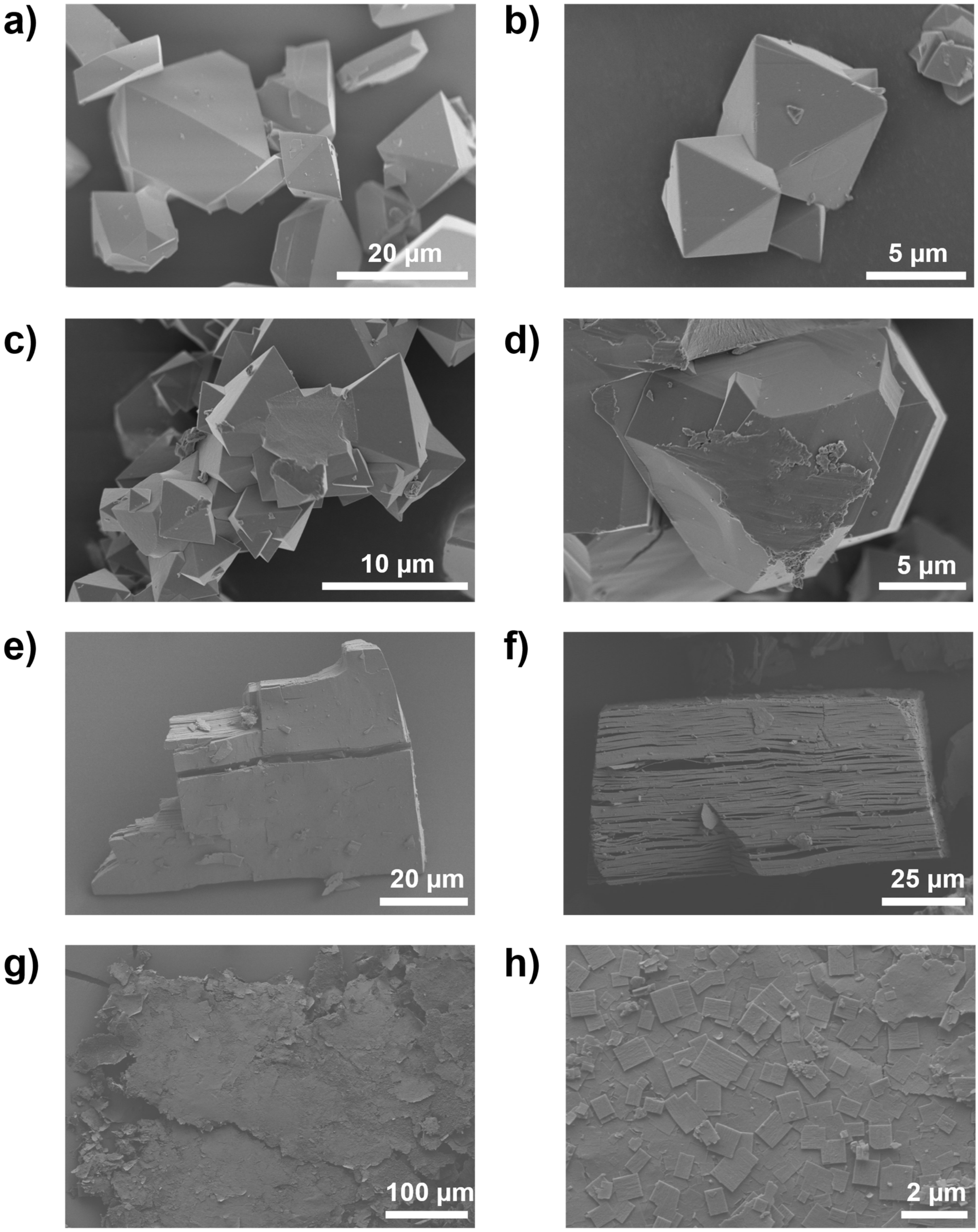

Figure S18. SEM images of: a) and b) HKUST-1; c) and d) HKUST-1 after H_2S treatment; e) and f) DUT-134 bulk after H_2S treatment; g) and h) DUT-134 carpets after H_2S treatment.

4.8.7 Raman spectroscopy after H_2S treatment

To proof a possible (poly)sulfide formation, the Raman spectra of the bulk and carpets were collected for the samples subjected to H_2S. After H_2S treatment a decrease in the intensities corresponding to Cu-O vibrations (375 - 380 cm^{-1}) could be observed, but peaks in the finger print region are preserved. Unfortunately, the peak, characteristic for CuS, at 474 cm^{-1} could not be used for identification, since the MOF itself has a strong signal centered at 477 cm^{-1}.

S-S vibrations in the 350 - 520 cm^{-1} region, which were observed and published by Ibarra and coworkers[6] for MFM-300 MOF after contact to H_2S and assigned to the polysulfides are not present in case of DUT-134. Only weak additional signals at 365 cm^{-1} and 471 cm^{-1} are visible. In addition, three new bands were identified and could be possibly assigned to polysulfides species. The peaks observed around 741 cm^{-1} and 539 cm^{-1} could correspond to S_x^{2-} (x = 4 - 8) and $S_3^{\bullet -}$.[7] The signal at 660 cm^{-1} could be potentially assigned to δ_S (S=O) vibration,[8] as the formation of oxygen containing sulfur compounds is not unlikely under ambient conditions.

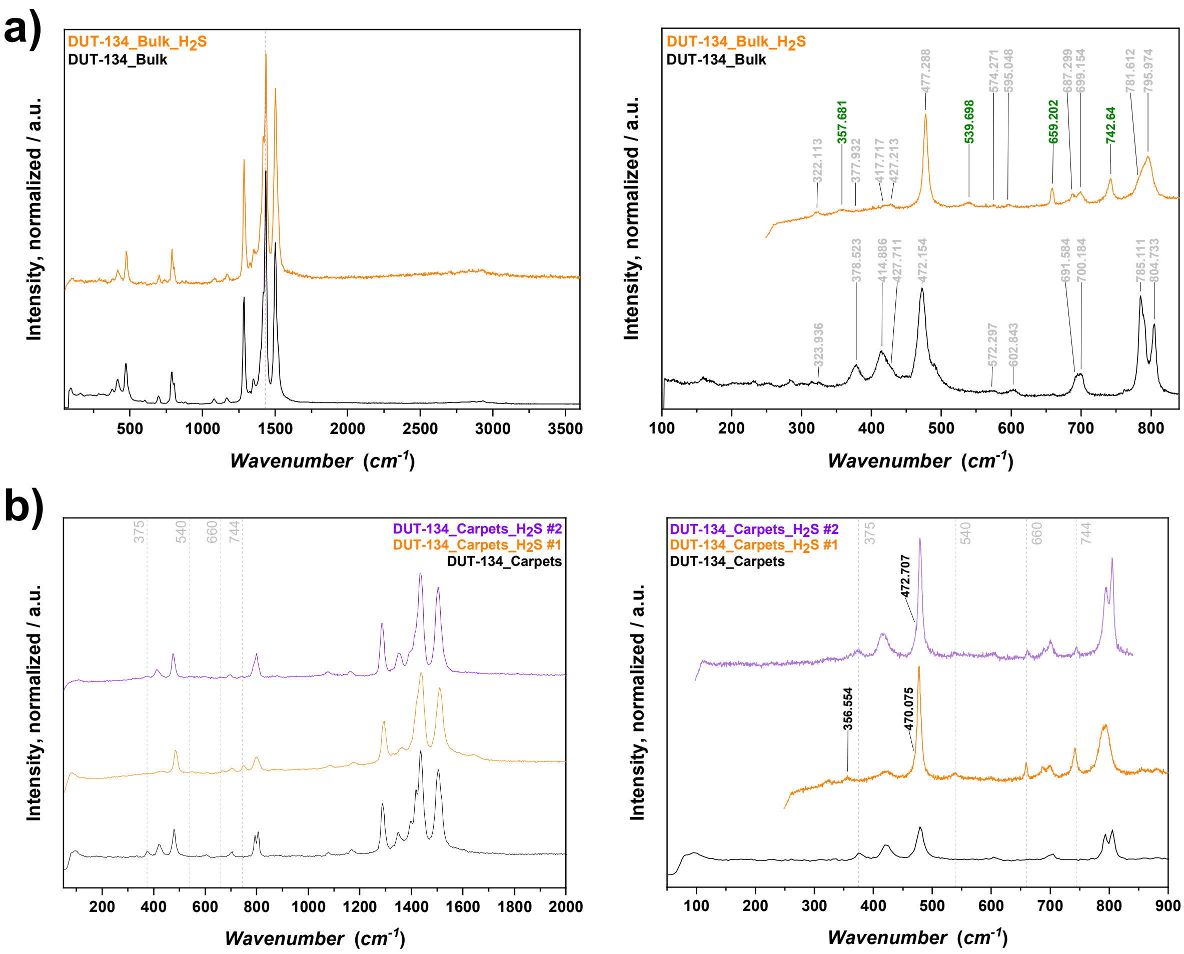

Figure S19. Raman spectra of: a) DUT-134(Cu) bulk before and after H_2S treatment; and b) two different positions on the surface of DUT-134(Cu) carpets after H_2S treatment in comparison to the spectrum of starting material.

4.9 Atomic Force Microscopy (AFM)

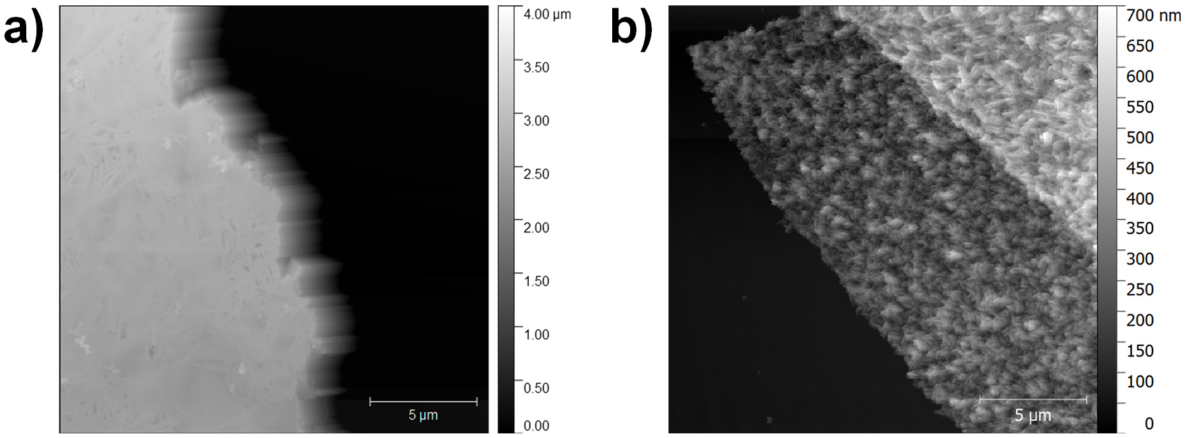

Figure S20. AFM images of: a) DUT-134(Cu) carpet cross-section with a maximum height of 3 - 4 µm; b) Edge of an already rolled-up carpet with a thickness of 5 - 10 µm.

References

[1] J. Frey, S. Proemmel, M. A. Armitage, A. B. Holmes, *Org. Synth.* **2006**, *83*, 209.
[2] F. Schwotzer, I. Senkovska, V. Bon, S. Lochmann, J. D. Evans, D. Pohl, B. Rellinghaus, S. Kaskel, *Inorg. Chem. Front.* **2021**, *8*, 3308.
[3] T. Rodenas, I. Luz, G. Prieto, B. Seoane, H. Miro, A. Corma, F. Kapteijn, F. X. Llabrés I Xamena, J. Gascon, *Nat. Mater.* **2015**, *14*, 48.
[4] A. Schneemann, R. Dong, F. Schwotzer, H. Zhong, I. Senkovska, X. Feng, S. Kaskel, *Chem. Sci.* **2021**, *12*, 1600.
[5] F. Sandra, N. Klein, M. Leistner, M. R. Lohe, M. Benusch, M. Woellner, J. Grothe, S. Kaskel, *Ind. Eng. Chem. Res.* **2015**, *54*, 6677.
[6] J. G. Flores, J. A. Zárate-Colín, E. Sánchez-González, J. R. Valenzuela, A. Gutiérrez-Alejandre, J. Ramírez, V. Jancik, J. Aguilar-Pliego, M. C. Zorrilla, H. A. Lara-García et al., *ACS Appl. Mater. Interfaces* **2020**, *12*, 18885.
[7] J.-T. Yeon, J.-Y. Jang, J.-G. Han, J. Cho, K. T. Lee, N.-S. Choi, *J. Electrochem. Soc.* **2012**, *159*, A1308.
[8] a) Y. Diao, K. Xie, S. Xiong, X. Hong, *J. Electrochem. Soc.* **2012**, *159*, A1816; b) L. Rintoul, K. Crawford, H. F. Shurvell, P. M. Fredericks, *Vib. Spectrosc.* **1997**, *15*, 171.
